# Tumor budding and fibrotic focus—proposed grading system for tumor budding in invasive carcinoma no special type of the breast

**DOI:** 10.1007/s00428-022-03337-0

**Published:** 2022-06-13

**Authors:** Miyuki Hiratsuka, Takahiro Hasebe, Yuki Ichinose, Ayaka Sakakibara, Akihiro Fujimoto, Noriko Wakui, Satomi Shibasaki, Masataka Hirasaki, Masanori Yasuda, Akemi Nukui, Hiroko Shimada, Hideki Yokogawa, Kazuo Matsuura, Takashi Hojo, Akihiko Osaki, Toshiaki Saeki

**Affiliations:** 1 Department of Breast Oncology, Saitama Medical University International Medical Center, 1397-1, Yamane, Hidaka City, Saitama 350-1298 Japan; 2 Community Health Science Center, Saitama Medical University, 29, Morohongou, Moroyama Town, Iruma district, Saitama 350-0495 Japan; 3 Department of Clinical Cancer Genomics, Saitama Medical University International Medical Center, 1397-1, Yamane, Hidaka City, Saitama 350-1298 Japan; 4 Department of Pathology, Saitama Medical University International Medical Center, 1397-1, Yamane, Hidaka City, Saitama 350-1298 Japan

**Keywords:** Tumor budding, Fibrotic focus, Tumor cell–stromal cell interaction, Tumor stroma, Breast cancer

## Abstract

Tumor budding grade is a very useful histological prognostic indicator for colorectal cancer patients. Recently, it has been also reported as a significant prognostic indicator in invasive breast carcinoma patients. Our group and others have previously reported that the presence of a fibrotic focus in the tumor is a very useful histological finding for accurately predicting the prognosis in patients with invasive carcinoma of no special type (ICNST) of the breast. The purpose of the present study was to investigate whether a grading system incorporating tumor budding in a fibrotic focus is superior to the conventional grading system for tumor budding to accurately predict outcomes in patients with ICNST. According to our new grading system, we classified the tumors into grade I (164 cases), grade II (581 cases), and grade III (110 cases), and the results clearly demonstrated the significant superiority of the new grading system over that of conventional tumor budding alone for accurately predicting outcomes in patients with ICNST. Our findings strongly suggest that tumor cells and tumor-stromal cells interaction play very important roles in tumor progression rather than tumor cells alone.

**Supplementary Information:**

The online version contains supplementary material available at 10.1007/s00428-022-03337-0.

## Introduction

Tumor budding (TB) refers to the small clusters of dedifferentiated tumor cells at the invasive margin of a tumor, and tumor budding grade (TBG) is very useful histological prognostic indicator in patients with colorectal cancer [1–4], and recently, TBG has also been reported as a significant prognostic indicator in patients with invasive breast carcinoma [5–7].

Our group and others have previously reported that the presence of a fibrotic focus (FF) is a very useful histological finding for accurately predicting the outcome in patients with invasive carcinoma of no special type (ICNST) of the breast [8–18]. The characteristics of tumor-stromal fibroblasts forming an FF and a high tumor angiogenesis ratio have been suggested to heighten the malignant potential of ICNSTs with an FF [19, 20]; other reports have indicated that the presence of FF is clearly associated with an intratumoral hypoxic condition of ICNSTs of the breast [10, 14, 21]. Furthermore, a cDNA microarray analysis reported previously clearly demonstrated specific biological characteristics of ICNSTs with an FF [12].

The purpose of the present study was to investigate whether a grading system for tumor budding incorporating both the conventional TBG and TBG in an FF proposed by us might be superior to the conventional TBG for accurately predicting the outcomes in patients with ICNSTs of the breast.

## Materials and methods

### Patients and histological examinations

The subjects of this study were 855 consecutive patients with ICNST of the breast who had undergone surgical treatment without prior neoadjuvant therapy at the Saitama Medical University International Medical Center between April 2007 and December 2015. All the patients were Japanese women, ranging in age from 29 to 92 years (median, 56 years). Of the 855 patients, 588 had undergone partial mastectomy, 261 had undergone modified radical mastectomy, and the remaining 6 had undergone standard radical mastectomy. Sentinel node dissection alone had been performed in 579 patients, and both sentinel node plus non-sentinel node dissection had been performed in 276 patients. None of the patients had received radiotherapy or chemotherapy before surgery, but 833 patients had received postoperative adjuvant therapy. The adjuvant therapy in these patients consisted of endocrine therapy in 413 patients, chemotherapy in 131 patients, chemoendocrine therapy in 211 patients, and trastuzumab with an endocrine therapy regimen and a chemotherapy regimen in 78 patients. All the tumors were classified according to the pathological UICC-TNM (pTNM) classification [22]. The protocol for this study was reviewed by the institutional review board of the Saitama Medical University International Medical Center.

**Fig. 1:**
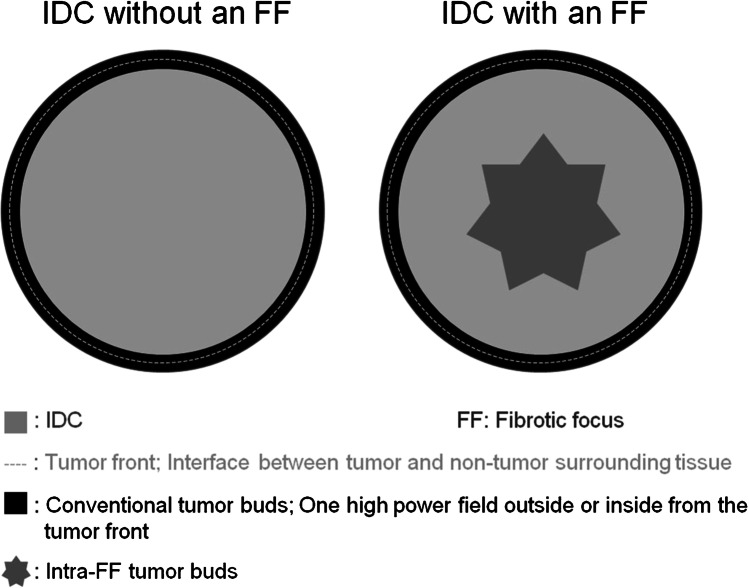
Schema of grading of peripheral tumor budding, intratumoral tumor budding, and tumor budding in a fibrotic focus

**Fig. 2:**
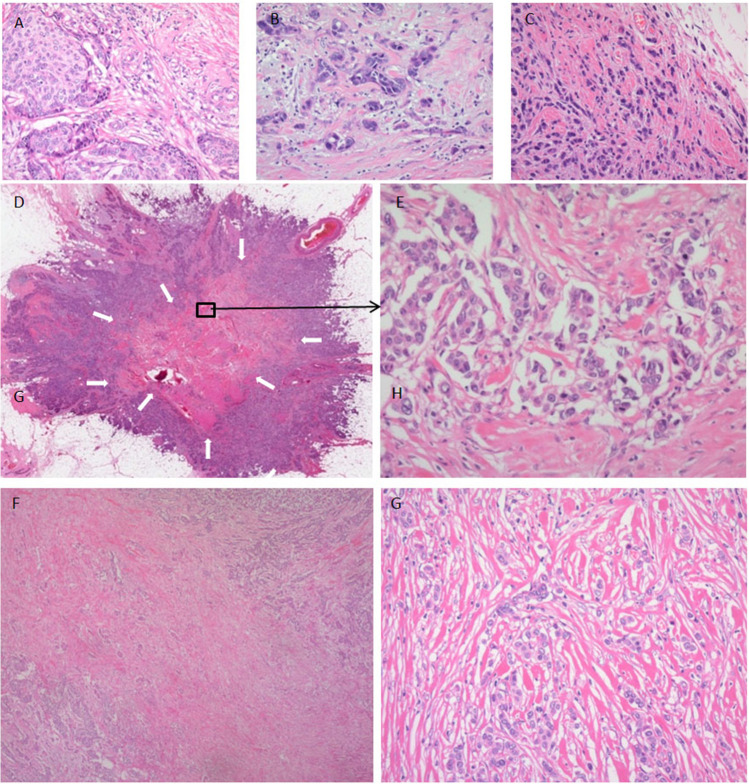
(**A**) Peripheral tumor budding grade 1 tumor cells. (**B**) Peripheral tumor budding grade 2 tumor cells. (**C**) Peripheral tumor budding grade 3 tumor cells. (**D–G**) Tumor budding in a fibrotic focus. (**D**) Fibrotic focus is indicated by arrows. (**F**) Invasive carcinoma no special type with an fibrotic focus. (**E**) Intra-tumor budding grade 1 tumor cells in an fibrotic focus. (**G**) Intra-tumor budding grade 3 tumor cells in an fibrotic focus. (**D–G**)

For the pathological examination of the tumors, the surgically resected specimens were fixed in 10% formalin. Well-known clinicopathological factors and the degree of infiltration by tumor-infiltrating lymphocytes (TILs; %) (Supplementary Table 1), conventional TBG, and presence/absence of an FF were evaluated (Supplementary Table 1). The percentage of TILs was counted in the stromal compartment (stromal TILs; magnification × 200–400), excluding the TILs outside the tumor border and around ductal carcinoma in situ and/or normal lobules [23–25]. All mononuclear cells (including lymphocytes and plasma cells) were counted, while polymorphonuclear leukocytes were excluded. The denominator used to determine the % stromal TILs is the area of stromal tissue, and a full assessment of the average number of TILs in the tumor area was used, without focusing only on hotspots. In the present study, the optimal cut-off value of the TIL (%) for accurately predicting the patient outcome was examined by univariate analysis using the Cox proportional hazards regression model, and the following were determined as potential cut-off values: 0%, 0–19%, and > 19% (Supplementary Table 1). Conventional TBG (CTBG) was determined by examination of peripheral area of the tumor grade (Fig. 1) [5–7]. CTBG was scored based on examination of the tumor buds at the invasive front of the tumor within 1.1 mm (2 × 1 high-power field) on either side of the tumor interface with normal tissue. TB was defined as an isolated single tumor cell or a cluster composed of fewer than five tumor cells at invasive front area, and was graded according to the three categories [1–7]. At first, two breast pathologists (MH and TH) examined H&E-stained sections at low-power magnification (× 4 or × 10) to identify five areas each of the tumor showing the highest density of TB (hot-spot) that were suitable for examining CTBG; then, the tumor buds were counted in these five spots at × 200 magnification (Zeiss Axioskop 40, field size 0.98 mm^2^) (Fig. 2A–C). The maximum tumor bud count in the five hot-spots for CTB was evaluated for each case [26]. In addition, the tumor buds in the FF were also examined in cases with an FF. Briefly, an FF is surrounded by a highly cellular zone of infiltrating carcinoma cells and occupies a variable percentages of the tumor area (Fig. 2D, F) [8, 9]. The maximum tumor bud count in five areas within an FF showing the highest density of tumor bud (hot-spots) were evaluated in cases with an FF (Fig. 2D–G). Fundamentally, TB was evaluated in H&E staining [26], but immunohistochemistry for E-cadherin (Flex monoclonal mouse anti-human E-cadherin, clone NCH38, ready-to-use; DAKO, CA, USA) was performed in all cases for confirming TB cells in each case and differentiating INST from lobular carcinoma. We defined the estrogen receptor status and progesterone receptor status of the tumor cells according to the ASCO/CAP guideline [27]. Cases positive immunostaining 1 to 100% of the tumor cell nuclei for ER or PgR were interpreted as showing a positive receptor (ER- and PgR-positive, respectively) status, while cases with positive staining of < 1% or 0% of the cell nuclei were considered as being negative for ER/ PgR expression. HER2 expression in the tumor cells was also categorized according to the ASCO/CAP guideline [28–30] (Supplementary Table 2). The Ki-67 (MIB-1, mouse monoclonal, ready-to-use; DAKO, Glostrup, Denmark) labeling index of stroma-invasive tumor cells was calculated as the percentage of tumor cells showing positive nuclear staining for Ki-67 among all the tumor cells counted. The fields for cell counting were selected randomly in the tumor area, and hot-spots of Ki-67-positive tumor cells were selected for assessing the Ki-67 labeling index; within this area, all tumor cells in each high-power field (× 400) were examined, and at least 500 tumor cells in each tumor were counted. The Ki-67 labeling index of stroma-invasive tumor cells was set at a threshold of 20% [31].

### Statistical analysis and patient outcome

Survival was evaluated over a median follow-up period of 58.0 months (range: 1.8 to 149.0 months) until March 2019. Tumor recurrence, local recurrence (breast skin), distant-organ metastasis (bone: 15 cases; lung: 10 cases; liver: 8 cases; distant lymph node: 9 cases; brain: 1; stomach: 1; multiple organs, e.g., bone/lung, bone/liver: 18 cases) and tumor-related death occurred in 79, 17, 62, and 26, respectively, of the 855 patients with ICNST enrolled in this study. Univariate and multivariate analyses were performed using the Cox proportional hazard regression model to identify the outcome predictive power of each factor. Disease-free survival curves, local recurrence, distant-organ metastasis and tumor-related death survival curves were drawn using the Kaplan–Meier method. For analyzing the risk factors for tumor recurrence, since the luminal B/HER2-positive group and HER2-positive group had less than 10 cases with tumor recurrence (nine cases in the former group and eight cases in the latter group) each other, the two groups were combined for the analysis. In regard to analysis of the risk factors for local recurrence, 10 or more cases of local recurrence were observed in each of the following groups: (1) overall cases; (2) cases aged > 39 years; (3) cases with a Ki-67 labeling index of > 20%; (4) cases with histological grade 3 (Supplementary Table 1). Therefore, we analyzed the risk factors for local recurrence in each of these groups. Similar analysis of the risk factors for distant-organ metastasis and/or tumor-related death could not be performed in all the groups, as there were < 10 cases of distant-organ metastasis and/or tumor-related death some of the groups.

## Results

### Prognostic power of conventional tumor budding grade

**Fig. 3:**
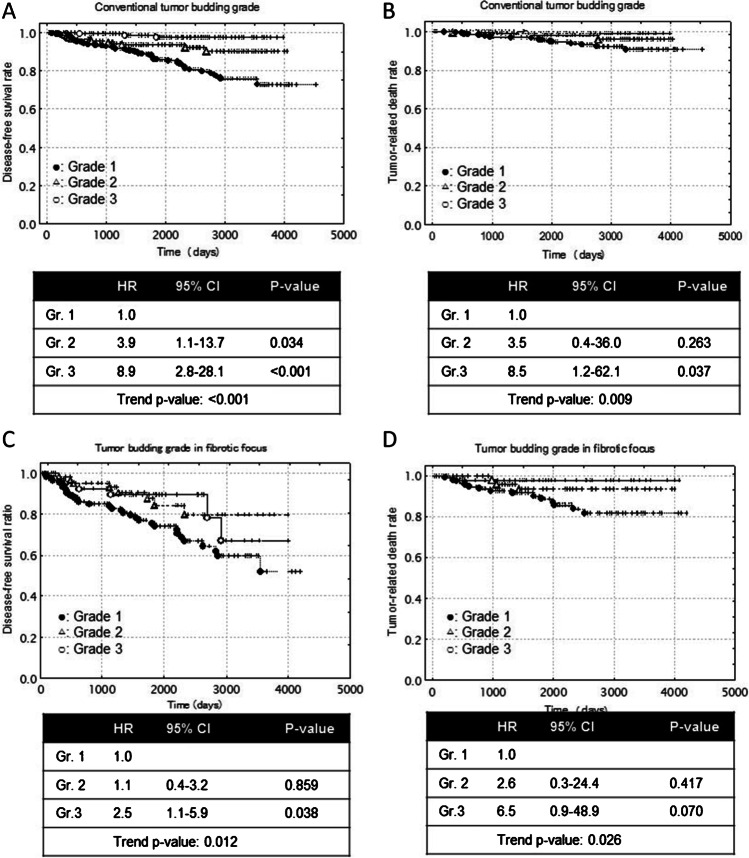
(**A**, **B**) Disease-free survival and tumor-related death survival periods decreased significantly with increasing peripheral tumor budding grade. (**C**, **D**) Disease-free survival and tumor-related death survival periods decreased significantly with increasing tumor budding grade in a fibrotic focus. HR, hazard ratio; CI, confidence interval; Gr., grade

Univariate analyses clearly demonstrated that progressive increase of the CTBG and of the TBG in the FF were associated with an increased risk of tumor recurrence, distant-organ metastasis, and tumor-related death, but not local recurrence (Table 1; Fig. 3A–D).

**Table 1:** Univariate analyses to determine the prognostic power of the conventional tumor budding grade and tumor budding grade in a fibrotic focus in cases of invasive carcinoma of no special type of the breast (overall)

Univariate analyses					
	Cases	TR (%)	LR (%)	DOM (%)	TRD (%)
	855	79	17	62	26
Conventional tumor budding grade					
Grade 1	183	3 (2)	1 (0.6)	2 (1)	1 (0.6)
Grade 2	208	13 (6)	3 (1)	10 (5)	4 (2)
Grade 3	464	63 (14)	13 (3)	50 (11)	21 (5)
*P* for trend		< 0.001	0.057	< 0.001	0.009
	246	48	9	38	19
Tumor budding grade in a fibrotic focus					
Grade 1	55	6 (11)	2 (4)	4 (7)	1 (2)
Grade 2	65	8 (12)	2 (3)	5 (9)	3 (5)
Grade 3	126	34 (27)	5 (4)	29 (23)	15 (12)
*P* for trend		0.012	0.824	0.008	0.026

Number of cases with a fibrotic focus was 246

TR, tumor recurrence; LR, local recurrence; DOM, distant-organ metastasis; TRD, tumor-related death

### Proposed system for tumor budding

**Fig. 4:**
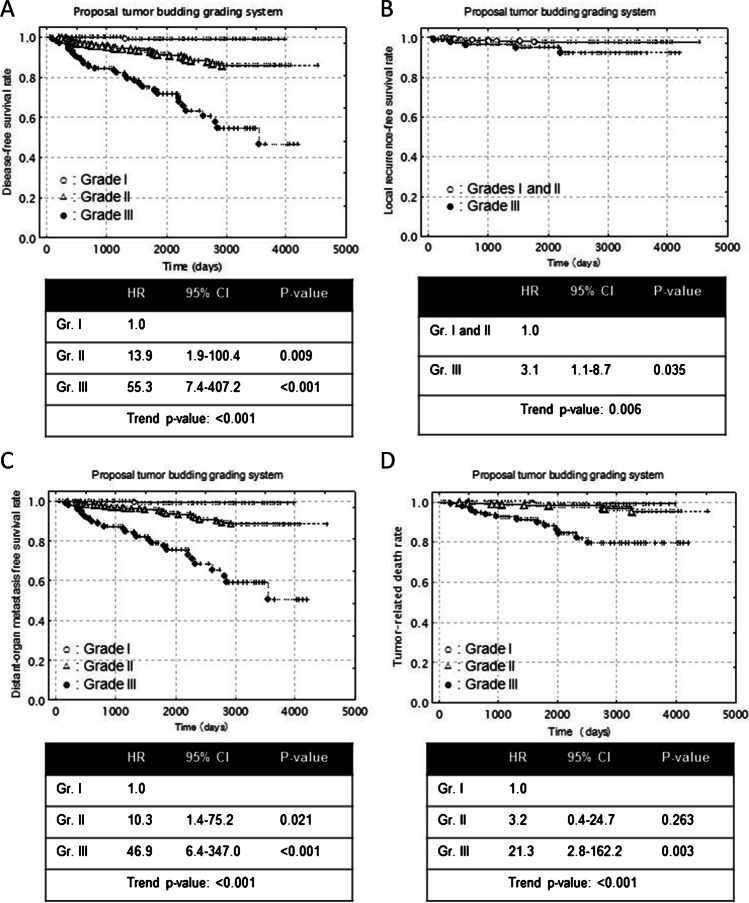
(**A**–**D**) Disease-free survival, local recurrence, distant-organ metastasis, and tumor-related death survival periods decreased significantly with increasing tumor budding grade according to the proposed grading system for tumor budding. HR, hazard ratio; CI, confidence interval; Gr., grade

Next, we attempted to develop a new grading system for tumor budding incorporating CTBG and the TBG in the FF in ICNSTs (Table 2). In cases without an FF, the CTBG was the final TB grade, while in cases with an FF, the TBG in the FF was added to the CTBG, e.g., in a case with an FF, CTB grade 2 and TB grade 2 in the FF were assigned a score of 4 (total TBG: 4) and finally classified into grade II of the proposed tumor budding grading system; in another case with an FF, CTB grade 3 and TB grade 3 in the FF were assigned a score of 6 (total TBG: 6) and finally classified into grade III of the proposed tumor budding grading system. The total TBG was classified into score 1 to 6; according to the results of univariate analysis performed to identify the predictors of tumor recurrence and tumor-related death, the score classes in the proposed tumor budding grading system (ProTBGS) were re-graded into grade I, grade II, and grade III (Table 2; Fig. 4A–D).

**Table 2:** Grading according to our proposed grading system for tumor budding in invasive carcinoma of no special type (overall)

Cases without an FF (609 cases)				Cases with an FF (246 cases)					
CTB grade		Total TB grade score class		CTB grade		TB grade in an FF		Total TB grade score class	
**1**		**1**		**1**		**1**		**2**	
**2**		**2**		**2**		**2**		**3**	
**3**		**3**		**3**		**3**		**4**	
								**5**	
								**6**	
**Score classes of CTB + TB grade in an FF**									
**Score**	**Cases**		**TR (%)**		***P* values**		**TRD (%)**		***P* values**
	855		79				26		
1	164		1 (0.6)				1 (0.6)		
2	178		7 (4)		0.036		1 (0.6)		0.998
3	298		26 (9)		0.052		5 (2)		0.287
4	45		8 (18)		0.088		3 (7)		0.061
5	60		5 (8)		0.180		2 (3)		0.151
6	110		32 (29)		0.002		14 (13)		0.056
Score class 1: cases without an FF, CTB grade 1 Score classes 2, 3, 4, and 5: cases without an FF, CTB grade 2 or 3; cases with an FF, CTB grade 1 and TB grade 1–3 in an FF, CTB grade 2 and TB grade 1–3 in an FF, CTB grade 3 and TB grade 1 or 2 in an FF Score class 6: cases with an FF, CTB grade 3 and TB grade 3 in an FF									
**Proposed tumor budding grading system**									
	Cases		TR (%)		LR (%)		DOM (%)		TRD (%)
Grade I	164		1 (0.6)		0		1 (0.6)		1 (0.6)
Grade II	581		46 (8)		12 (2)		34 (6)		11 (2)
Grade III	110		32 (29)		5 (5)		27 (25)		14 (13)
*P* for trend			< 0.001		0.006		< 0.001		< 0.001
Grade I: score class 1 Grade II: score classes 2, 3, 4, and 5 Grade III: score class 6									

CTB, conventional tumor budding; FF, fibrotic focus; TR, tumor recurrence; LR, local recurrence; DOM, distant-organ metastasis; TRD, tumor-related death

### Prognostic power of the proposed tumor budding grading system

The abilities of the CTBG and ProTBGS to predict the clinical outcome were evaluated separately, along with those of well-known clinicopathological factors and tumor-infiltrating lymphocytes (%) (Supplementary Table 1) using model 1 (CTBG) and model 2 (ProTBGS), respectively.

Multivariate analysis using model 1 identified CTBG grade 3 as being associated with significantly increased hazard ratios for tumor recurrence and distant-organ metastasis, but not for local recurrence or tumor-related death (Table 3). Presence of an FF and presence of muscle invasion were significantly associated with tumor recurrence, distant-organ metastasis, and tumor-related death (Table 3). Histological grade 3 was significantly associated with local recurrence, distant-organ metastasis, and tumor-related death (Table 3). Multivariate analyses using model 2 identified ProTBGS grade III as being associated with significantly increased hazard ratios (as high as the presence of muscle invasion) for tumor recurrence, distant-organ metastasis, and tumor-related death (Table 3). Histological grade 3 was significantly associated with local recurrence and tumor-related death (Table 3).

**Table 3:** Multivariate analyses to identify predictors of the clinical outcomes in patients with invasive carcinoma of no special type of the breast (overall)

	Cases		No. of patients (%)												
			TR				LR				DOM			TRD	
			+		HR 95% CI *P* value		+		HR 95% CI *P* value		+		HR 95% CI *P* value	+	HR 95% CI *P* value
	855		79 (9)				17 (2)				62 (7)			26 (3)	
**Model 1**															
Conventional tumor budding grade															
Grade 1	183		3 (2)		1.0		1 (0.6)		1.0		2 (1)		1.0	1 (0.6)	1.0
Grade 2	208		13 (6)		3.6 0.9–13.2 0.052		3 (1)		3.7 0.4–39.3 0.271		10 (5)		3.9 0.8–19.1 0.092	4 (2)	1.8 0.2–19.5 0.620
Grade 3	464		63 (14)		5.4 1.6–18.3 0.006		13 (3)		5.1 0.6–41.6 0.130		50 (11)		6.0 1.4–26.8 0.019	21 (5)	3.7 0.5–30.2 0.221
Fibrotic focus															
Absent	609		31 (5)		1.0		8 (1)		1.0		23 (4)		1.0	1 (0.6)	1.0
Present	246		48 (20)		2.8 1.3–3.6 0.005		9 (4)		1.5 0.5–4.5 0.449		39 (16)		2.4 1.3–4.3 0.006	4 (2)	4.5 1.8–11.0 0.001
Muscle invasion															
Absent	845		75 (9)		1.0		17 (2)		1.0		58 (7)		1.0	23 (3)	1.0
Present	10		4 (40)		3.5 1.1–10.8 0.034		0		NA		4 (40)		3.9 1.2–13.5 0.029	3 (30)	7.0 1.8–27.7 0.006
Radiotherapy															
No	469		54 (12)		1.0		13 (3)		1.0		41 (9)		1.0	18 (4)	1.0
Yes	386		25 (7)		0.5 0.3–0.9 0.016		4 (1)		0.3 0.1–0.9 0.045		21 (5)		0.5 0.3–0.9 0.027	8 (2)	0.4 0.1–1.0 0.058
UICC pN category															
pN0	610		35 (6)		1.0		10 (2)		1.0		25 (4)		1.0	9 (2)	1.0
pN1	163		25 (15)		1.9 0.7–5.0 0.210		4 (3)		0.9 0.3–2.8 0.826		21 (13)		2.3 0.8–6.8 0.152	7 (4)	1.7 0.3–9.2 0.515
pN2	54		10 (19)		1.9 0.6–6.5 0.290		0		NA		10 (19)		2.7 0.7–10.3 0.155	6 (11)	2.9 1.0–7.9 0.042
pN3	28		9 (32)		3.8 1.1–12.8 0.030		3 (11)		7.3 2.0–26.7 0.003		6 (21)		3.8 0.9–15.8 0.067	4 (14)	3.4 1.1–10.7 0.035
Histological grade															
Grade 1	264		10 (4)		1.0		2 (0.8)		1.0		8 (3)		1.0	2 (0.8)	1.0
Grade 2	352		25 (7)		0.8 0.4–1.8 0.646		4 (1)		0.8 0.1–4.8 0.834		21 (6)		0.8 0.3–2.0 0.655	4 (1)	0.4 0.06–2.6 0.350
Grade 3	239		44 (18)		1.3 0.6–3.1 0.554		11 (5)		4.1 1.5–11.1 0.006		33 (14)		2.3 1.4–3.9 0.002	20 (8)	3.7 1.3–10.4 0.013
Age (years)															
≤ 39	59		12 (20)		1.0		1 (2)		1.0		11 (19)		1.0	4 (7)	1.0
> 39	796		67 (8)		0.5 0.2–0.9 0.020		16 (2)		1.7 0.2–13.2 0.634		51 (6)		0.4 0.2–0.8 0.007	22 (3)	0.6 0.2–2.1 0.456
Adjuvant therapy															
No	22		5 (23)		1.0		1 (5)		1.0		4 (18)		1.0	1 (5)	1.0
Yes	833		74 (9)		0.1 0.03–0.3 < 0.001		16 (2)		0.4 0.4–3.6 0.405		58 (7)		0.06 0.02–0.2 < 0.001	25 (3)	0.2 0.02–2.0 0.151
Ki-67 labeling index															
< 20	420		20 (5)		1.0		3 (0.7)		1.0		17 (4)		1.0	7 (2)	1.0
≥ 20	435		59 (14)		1.9 1.0–3.4 0.045		14 (3)		2.9 0.7–11.4 0.134		45 (10)		1.5 0.3–3.0 0.210	19 (4)	0.6 0.2–1.9 0.379
Hormone receptor status															
Negative	141		21 (15)		1.0		6 (4)		1.0		15 (11)		1.0	12 (1)	1.0
Positive	714		58 (8)		0.7 0.3–1.4 0.296		11 (2)		0.6 0.2–2.0 0.377		47 (7)		0.8 0.04–1.6 0.505	14 (2)	0.2 0.08–0.6 0.002
Perineural invasion															
Absent	702		58 (8)		1.0		14 (2)		1.0		44 (6)		1.0	13 (2)	1.0
Present	153		21 (14)		1.2 0.7–2.1 0.572		3 (2)		0.6 0.1–2.6 0.494		18 (12)		1.4 0.7–2.6 0.366	13 (9)	4.5 1.8–11.0 0.001
**Model 2**															
Proposed tumor budding grading system															
Grade I	164	1 (0.6)		1.0		0		1.0		1 (0.6)		1.0		1 (0.6)	1.0
Grade II	581	46 (8)		12.2 1.6–91.7 0.016		12 (2)		1.0		34 (6)		9.1 1.2–71.8 0.036		11 (2)	1.3 0.2–11.0 0.814
Grade III	110	32 (29)		33.7 4.2–264.4 < 0.001		5 (5)		2.1 0.7–6.9 0.212		27 (25)		28.6 3.4–241.4 0.002		14 (13)	6.8 2.9–16.2 < 0.001
Muscle invasion															
Absent	845	75 (9)		1.0		17 (2)		1.0		58 (7)		1.0		23 (3)	1.0
Present	10	4 (40)		3.9 1.3–12.1 0.019		0		NA		4 (40)		4.5 1.3–15.3 0.015		3 (30)	9.6 2.4–37.8 0.001
Adjuvant therapy															
No	22	5 (23)		1.0		1 (5)		1.0		4 (18)		1.0		1 (5)	1.0
Yes	833	74 (9)		0.1 0.02–0.4 < 0.001		16 (2)		0.4 0.04–3.3 0.378		58 (7)		0.08 0.02–0.3 < 0.001		25 (3)	0.2 0.02–2.0 0.187
Age (years)															
≤ 39	59	12 (20)		1.0		1 (2)		1.0		11 (19)		1.0		4 (7)	1.0
> 39	796	67 (8)		0.5 0.3–0.9 0.036		16 (2)		1.3 0.2–10.6 0.794		51 (6)		0.4 0.2–0.8 0.014		22 (3)	0.8 0.2–2.8 0.750
Tumor-infiltrating lymphocytes (%)															
0	6	1 (17)		1.0		0		1.0		1 (17)		1.0		0	1.0
1–19	725	72 (10)		0.2 0.03–1.8 0.154		14 (2)		1.0		58 (8)		0.2 0.02–1.9 0.158		25 (4)	1.0
> 19	124	6 (5)		0.08 0.008–0.8 0.029		3 (2)		0.9 0.2–3.7 0.899		3 (2)		0.05 0.004–0.5 0.015		1 (0.8)	0.1 0.01–1.0 0.052
Histological grade															
Grade 1	264	10 (4)		1.0		2 (0.8)		1.0		8 (3)		1.0		2 (0.8)	1.0
Grade 2	352	25 (7)		0.9 0.4–1.9 0.734		4 (1)		0.8 0.1–4.8 0.834		21 (6)		0.9 0.4–2.1 0.768		4 (1)	0.5 0.07–3.3 0.471
Grade 3	239	44 (18)		1.4 0.6–3.2 0.479		11 (5)		4.1 1.5–11.1 0.006		33 (14)		1.3 0.5–3.2 0.645		20 (8)	3.4 1.3–9.0 0.015
Invasive tumor size (mm)															
≤ 20	326	13 (4)		1.0		5 (2)		1.0		8 (3)		1.0		1 (0.3)	1.0
> 20 to ≤ 50	483	52 (11)		1.3 0.6–2.6 0.479		9 (2)		0.7 0.2–2.3 0.529		43 (9)		1.8 0.8–4.1 0.189		18 (4)	4.9 0.6–42.3 0.139
> 50	46	14 (30)		2.2 0.8–5.9 0.100		3 (7)		1.4 0.2–9.6 0.725		11 (24)		3.6 1.1–11.1 0.028		7 (15)	3.2 1.3–7.9 0.014
Radiotherapy															
No	469	54 (12)		1.0		13 (3)		1.0		41 (9)		1.0		18 (4)	1.0
Yes	386	25 (7)		0.5 0.3–0.9 0.023		4 (1)		0.4 0.1–1.2 0.085		21 (5)		0.6 0.3–1.1 0.091		8 (2)	0.4 0.2–1.2 0.105
Ki-67 labeling index															
< 20	420	20 (5)		1.0		3 (0.7)		1.0		17 (4)		1.0		7 (2)	1.0
≥ 20	435	59 (14)		1.9 1.0–3.5 0.041		14 (3)		2.9 0.7–11.4 0.140		45 (10)		1.6 0.8–3.1 0.207		19 (4)	0.6 0.2–1.9 0.406
UICC pN category															
pN0	610	35 (6)		1.0		10 (2)		1.0		25 (4)		1.0		9 (2)	1.0
pN1	163	25 (15)		1.5 0.6–4.0 0.388		4 (3)		0.9 0.3–2.8 0.826		21 (13)		1.8 0.6–5.3 0.306		7 (4)	1.2 0.2–6.5 0.873
pN2	54	10 (19)		1.7 0.5–5.5 0.405		0		NA		10 (19)		2.3 0.6–8.5 0.235		6 (11)	3.3 0.4–26.0 0.265
pN3	28	9 (32)		2.5 0.7–8.6 0.149		3 (11)		7.3 2.0–26.7 0.003		6 (21)		2.3 0.5–9.9 0.253		4 (14)	2.2 0.2–20.4 0.485
Hormone receptor status															
Negative	141	21 (15)		1.0		6 (4)		1.0		15 (11)		1.0		12 (1)	1.0
Positive	714	58 (8)		0.6 0.3–1.2 0.162		11 (2)		0.6 0.2–2.3 0.449		47 (7)		0.7 0.3–1.4 0.279		14 (2)	0.2 0.06–0.4 < 0.001
Perineural invasion															
Absent	702	58 (8)		1.0		14 (2)		1.0		44 (6)		1.0		13 (2)	1.0
Present	153	21 (14)		1.1 0.6–2.0 0.709		3 (2)		0.6 0.1–2.6 0.493		18 (12)		1.3 0.7–2.4 0.469		13 (9)	3.8 1.6–9.1 0.003

HR, hazard ratio; CI, confidence interval; TR, tumor recurrence; LR, local recurrence; DOM, distant-organ metastasis; TRD, tumor-related death; + , present

Table 4 shows the factors that were found to be significantly associated with tumor recurrence and/or overall survival according to the UICC pTNM stages. In UICC pTNM stage I cases, analysis using model 1 identified CTBG grade 3 and a Ki-67 labeling index of ≧20% as being significantly associated with tumor recurrence, and analysis using model 2 identified ProTBGS grade III and a Ki-67 labeling index of ≧20% as being significantly associated with tumor recurrence. In UICC pTNM stage II, analysis using model 1 identified CTBG grade 3 as being associated with an increased hazard ratio for tumor recurrence, but not for distant-organ metastasis or tumor-related death; histological grade 3 was the only factor that was found to be associated with increased hazard ratios for tumor recurrence, distant-organ metastasis, and tumor-related death (Table 4). Analysis using model 2 identified ProTBGS grade III as the only factor associated with increased hazard ratios for tumor recurrence, distant-organ metastasis, and tumor-related death. In UICC pTNM stage III cases, analysis using model 1 identified hormone receptor status as the only factor significantly associated with tumor recurrence, distant-organ metastasis, and tumor-related death (Table 4). Analysis using model 1 failed to reveal any association between CTBG grade 3 and tumor recurrence, distant-organ metastasis, or tumor-related death; on the other hand, presence of an FF was associated with increased hazard ratios for distant-organ metastasis and tumor-related death (Table 4). Analysis using model 2 identified hormone receptor status as the only factor significantly associated with tumor recurrence, distant-organ metastasis, and tumor-related death (Table 4). Analysis using model 2 identified ProTBGS grade III as being significantly associated with tumor recurrence and distant-organ metastasis (Table 4).

**Table 4:** Multivariate analyses to identify predictors of the clinical outcomes in patients with invasive carcinoma of no special type of the breast according to the UICC pTNM stage

UICC pTNM stage I																		
**Tumor recurrence**																		
					Cases				TR (%)	HR			95% CI				*P* value	
					286				12 (4)									
**Model 1**																		
Conventional tumor budding grade																		
Grade 1					93				0	1.0								
Grade 2					85				3 (4)	1.0								
Grade 3					108				9 (8)	4.5			1.1–19.1				0.045	
Fibrotic focus																		
Absent					239				8 (3)	1.0								
Present					47				4 (9)	1.3			0.3–6.1				0.722	
Ki-67 labeling index																		
< 20					164				2 (1)	1.0								
≥ 20					122				10 (8)	7.1			1.2–42.3				0.032	
**Model 2**																		
Proposed tumor budding grading system																		
Grade I					88				0	1.0								
Grade II					186				10 (5)	1.0								
Grade III					12				2 (17)	7.1			1.2–44.8				0.038	
Ki-67 labeling index																		
< 20					164				2 (1)	1.0								
≥ 20					122				10 (8)	9.0			1.5–53.2				0.016	
**UICC pTNM stage II**																		
		Cases				No. of patients (%)												
						TR						DOM				TRD		
		435				39 (9)						31 (7)				12 (3)		
						Present		HR 95% CI *P* value				Present			HR 95% CI *P* value	Present		HR 95% CI *P* value
**Model 1**																		
Conventional tumor budding grade																		
Grade 1		83				2 (2)		1.0				2 (2)			1.0	1 (1)		1.0
Grade 2		108				7 (7)		2.9 0.001–14.8 0.213				5 (5)			1.9 0.3–10.6 0.485	2 (2)		1.3 0.1–14.6 0.855
Grade 3		244				30 (12)		2.3 1.0–4.9 0.033				24 (10)			2.7 0.6–13.3 0.234	9 (4)		1.7 0.4–6.8 0.456
Fibrotic focus																		
Absent		307				17 (6)		1.0				13 (4)			1.0	4 (1)		1.0
Present		128				22 (17)		2.4 1.3–4.6 0.010				18 (14)			3.0 1.4–6.6 0.005	8 (6)		2.0 0.5–7.9 0.316
Histological grade																		
Grade 1		119				5 (4)		1.0				4 (3)			1.0	1 (0.8)		1.0
Grade 2		179				12 (7)		1.3 0.4–4.4 0.646				10 (6)			1.3 0.4–4.4 0.646	0		1.0
Grade 3		137				22 (16)		2.1 1.1–4.0 0.027				17 (12)			2.4 1.1–5.6 0.033	11 (8)		17.4 2.1–160.1 0.010
Adjuvant therapy																		
No		9				2 (22)		1.0				2 (22)			1.0	0		1.0
Yes		426				37 (9)		0.2 0.04–0.7 0.015				29 (7)			0.06 0.01–0.3 < 0.001	12 (3)		NA
Blood vessel invasion																		
Absent		283				22 (8)		1.0				18 (6)			1.0	5 (2)		1.0
Present		152				17 (11)		2.2 1.1–4.9 0.037				13 (9)			2.1 0.9–4.8 0.095	7 (5)		4.6 1.2–17.0 0.025
Age (years)																		
≤ 39		37				7 (19)		1.0				7 (19)			1.0	2 (5)		1.0
> 39		398				32 (8)		0.4 0.2–1.1 0.060				24 (6)			0.3 0.1–0.7 0.006	10 (3)		0.9 0.2–4.8 0.996
**Model 2**																		
Proposed tumor budding grading system																		
Grade I		73				1 (1)		1.0				1 (1)			1.0	1 (1)		1.0
Grade II		308				24 (8)		6.5 0.8–55.3 0.081				19 (6)			4.3 0.6–32.4 0.156	5 (2)		1.0 0.1–9.2 0.998
Grade III		54				14 (26)		17.9 2.1–157.0 0.009				11 (20)			14.6 1.9–117.0 0.011	6 (11)		4.0 1.3–12.8 0.018
Adjuvant therapy																		
No		9				2 (22)		1.0				2 (22)			1.0	0		1.0
Yes		426				37 (9)		0.1 0.02–0.6 0.009				29 (7)			0.08 0.02–0.4 0.001	12 (3)		NA
Blood vessel invasion																		
Absent		283				22 (8)		1.0				18 (6)			1.0	5 (2)		1.0
Present		152				17 (11)		2.4 1.2–4.9 0.021				13 (9)			2.2 0.6–5.2 0.060	7 (5)		3.7 1.2–11.8 0.027
Age (years)																		
≤ 39		37				7 (19)		1.0				7 (19)			1.0	2 (5)		1.0
> 39		398				32 (8)		0.5 0.2–1.2 0.108				24 (6)			0.3 0.1–0.8 0.009	10 (3)		1.4 0.3–7.1 0.707
Histological grade																		
Grade 1		119				5 (4)		1.0				4 (3)			1.0	1 (0.8)		1.0
Grade 2		179				12 (7)		1.2 0.4–3.7 0.711				10 (6)			1.4 0.4–4.7 0.608	0		1.0
Grade 3		137				22 (16)		2.8 0.8–9.2 0.092				17 (12)			2.9 0.7–11.1 0.117	11 (8)		23.9 3.0–186.4 0.003
**UICC pTNM stage III**																		
	Cases			No. of patients (%)														
				TR							DOM					TRD		
	134			28 (21)							23 (17)					13 (10)		
				Present			HR 95% CI *P* value				Present			HR 95% CI *P* value		Present		HR 95% CI *P* value
**Model 1**																		
Conventional tumor budding grade																		
Grade 1	7			1 (14)			1.0				0				1.0	0		1.0
Grade 2	15			3 (20)			3.1 0.2–46.7 0.408				3 (20)				1.0	2 (13)		1.0
Grade 3	112			24 (21)			4.6 0.4–52.5 0.226				20 (18)				2.0 0.3–11.6 0.442	11 (8)		4.2 0.7–24.9 0.128
Fibrotic focus																		
Absent	63		6 (10)				1.0				4 (6)				1.0	2 (3)		1.0
Present	71		22 (31)				3.5 0.9–12.6 0.062				19 (27)				6.4 1.2–33.7 0.029	11 (16)		6.9 1.3–36.1 0.020
Hormone receptor status																		
Negative	28		10 (36)				1.0				8 (29)				1.0	5 (18)		1.0
Positive	106		18 (17)				0.07 0.02–0.2 < 0.001				15 (14)				0.06 0.01–0.3 < 0.001	8 (8)		0.08 0.02–0.3 < 0.001
Tumor-infiltrating lymphocytes (%)																		
0	1		1 (100)				1.0				1 (100)				1.0	0		1.0
1–19	116		26 (22)				0.02 0.0009–0.5 0.015				21 (18)				0.03 0.001–0.8 0.033	13 (11)		1.0
> 19	17		1 (6)				0.0004 0.000004–0.05 0.001				1 (6)				0.001 0.0001–0.1 0.003	0		NA
Adjuvant therapy																		
No	6		2 (33)				1.0				2 (33)				1.0	1 (17)		1.0
Yes	128		26 (20)				0.02 0.001–0.5 0.016				21 (16)				0.006 0.0002–0.2 0.005	12 (9)		0.7 0.05–12.1 0.820
Radiotherapy																		
No	68		19 (28)				1.0				15 (22)				1.0	9 (13)		1.0
Yes	66		9 (14)				0.3 0.09–0.9 0.037				8 (12)				0.5 0.1–1.5 0.211	4 (6)		0.3 0.06–1.4 0.136
Age (years)																		
≤ 39	8		4 (50)				1.0				4 (50)				1.0	2 (25)		1.0
> 39	126		24 (19)				0.2 0.03–1.0 0.051				19 (15)				0.1 0.02–0.9 0.047	11 (9)		0.3 0.01–6.9 0.440
Muscle invasion																		
Absent	126		24 (19)				1.0				19 (15)				1.0	10 (8)		1.0
Present	8		4 (50)				2.0 0.3–17.4 0.526				4 (50)				2.7 0.3–28.6 0.394	3 (38)		6.4 1.4–29.3 0.017
Perineural invasion																		
Absent	85		14 (16)				1.0				11 (13)				1.0	3 (4)		1.0
Present	49		14 (29)				2.8 0.9–8.1 0.071				12 (25)				3.5 0.9–13.4 0.066	10 (20)		6.8 1.7–26.9 0.006
**Model 2**																		
Proposed tumor budding grading system																		
Grade I	3		0				1.0				0				1.0	0		1.0
Grade II	87		12 (14)				1.0				8 (9)				1.0	5 (6)		1.0
Grade III	44		16 (36)				2.9 1.2–6.9 0.014				15 (34)				5.9 1.7–21.2 0.006	8 (18)		3.5 0.8–15.2 0.093
Hormone receptor status																		
Negative	28		10 (36)				1.0				8 (29)				1.0	5 (18)		1.0
Positive	106		18 (17)				0.2 0.07–0.4 < 0.001				15 (14)				0.07 0.02–0.4 < 0.001	8 (8)		0.1 0.04–0.5 0.004
Tumor-infiltrating lymphocytes (%)																		
0	1		1 (100)				1.0				1 (100)				1.0	0		1.0
1–19	116		26 (22)				0.1 0.01–0.9 0.044				21 (18)				0.01 0.004–0.3 0.010	13 (11)		1.0
> 19	17		1 (6)				0.002 0.0003–0.07 0.001				1 (6)				0.001 0.0006–0.5 0.001	0		NA
Adjuvant therapy																		
No	6		2 (33)				1.0				2 (33)				1.0	1 (17)		1.0
Yes	128		26 (20)				0.03 0.002–0.5 0.016				21 (16)				0.008 0.0003–0.2 0.005	12 (9)		0.4 0.02–7.7 0.545
Radiotherapy																		
No	68		19 (28)				1.0				15 (22)				1.0	9 (13)		1.0
Yes	66		9 (14)				0.3 0.1–0.9 0.022				8 (12)				0.5 0.1–1.6 0.243	4 (6)		0.2 0.06–0.9 0.036
Perineural invasion																		
Absent	85		14 (16)				1.0				11 (13)				1.0	3 (4)		1.0
Present	49		14 (29)				2.3 1.0–5.0 0.040				12 (25)				2.8 0.8–10.1 0.108	10 (20)		13.9 2.5–78.6 0.003
Age (years)																		
≤ 39	8		4 (50)				1.0				4 (50)				1.0	2 (25)		1.0
> 39	126		24 (19)				0.2 0.05–0.6 0.006				19 (15)				0.2 0.03–1.6 0.122	11 (9)		0.3 0.02–4.0 0.344
Histological grade																		
Grade 1	21		2 (10)				1.0				2 (10)				1.0	1 (5)		1.0
Grade 2	64		10 (16)				0.9 0.2–5.5 0.970				8 (13)				0.5 0.08–3.2 0.461	4 (6)		0.4 0.03–5.1 0.448
Grade 3	49		16 (33)				2.6 1.1–6.2 0.026				13 (27)				1.4 0.2–9.1 0.729	8 (16)		2.7 0.6–11.1 0.178

HR, hazard ratio; CI, confidence interval; NA, not available; TR, tumor recurrence; DOM, distant-organ metastasis; TRD, tumor-related death

Table 5 shows the factors that were found by multivariate analyses as being significantly associated with tumor recurrence and/or distant-organ metastasis, according to the intrinsic subtype of the tumor. Multivariate analyses using model 1 identified CTBG grade 3 as being associated with significantly increased hazard ratio for distant-organ metastasis only in cases with the luminal B/HER2-negative subtype of tumor (Table 5); multivariate analyses using model 2 clearly identified ProTBGS grade III as being associated with increased hazard ratios for tumor recurrence and distant-organ metastasis in patients with almost all intrinsic subtypes of tumor, except the basal-like subtype (Table 5).

**Table 5:** Multivariate analyses to identify factors predicting the clinical outcomes in patients with invasive carcinoma of no special type of the breast, according to the intrinsic tumor subtype

	Cases		No. of patients (%)							
**Luminal A subtype**										
			Tumor recurrence					Distant-organ metastasis		
			Present		HR; 95% CI *P* value			Present		HR; 95% CI *P* value
	334		13 (4)					12 (4)		
**Model 1**										
Conventional tumor budding grade										
Grade 1	75		0		1.0			0		1.0
Grade 2	81		1 (1)		1.0			1 (1)		1.0
Grade 3	178		12 (7)		7.2; 0.9–59.3 0.070			11 (6)		0.3; 0.4–28.8 0.288
Fibrotic focus										
Absent	266		4 (2)		1.0			4 (2)		1.0
Present	68		9 (13)		10.0; 3.0–33.6 < 0.001			8 (12)		8.7; 2.4–31.6 0.001
Age (years)										
≤ 39	16		3 (19)		1.0			3 (19)		1.0
> 39	318		10 (3)		0.1; 0.03–0.4 0.001			9 (3)		0.07; 0.02–0.3 < 0.001
Muscle invasion										
Absent	328		12 (4)		1.0			11 (3)		1.0
Present	6		1 (17)		18.8; 1.7–194.7 0.016			1 (17)		39.0; 3.4–27.3 0.004
Radiotherapy										
No	173		10 (6)		1.0			9 (5)		1.0
Yes	161		3 (2)		0.2; 0.05–0.9 0.037			3 (2)		0.1; 0.02–0.6 0.012
**Model 2**										
Proposed tumor budding grading system										
Grade I	69		0		1.0			0		1.0
Grade II	230		6 (3)		1.0			6 (3)		1.0
Grade III	35		7 (20)		8.7; 2.8–27.0 < 0.001			6 (17)		7.1; 2.1–23.5 0.001
Age (years)										
≤ 39	16		3 (19)		1.0			3 (19)		1.0
> 39	318		10 (3)		0.1; 0.03–0.4 0.001			9 (3)		0.09; 0.02–0.4 0.001
Muscle invasion										
Absent	328		12 (4)		1.0			11 (3)		1.0
Present	6		1 (17)		21.2; 1.8–247.2 0.015			1 (17)		22.1; 1.9–254.7 0.013
Radiotherapy										
No	173		10 (6)		1.0			9 (5)		1.0
Yes	161		3 (2)		0.2; 0.03–0.7 0.021			3 (2)		0.2; 0.03–0.8 0.028
**Luminal B/HER2-negative subtype**										
			Tumor recurrence					Distant-organ metastasis		
			Present		HR; 95% CI *P* value			Present		HR; 95% CI *P* value
	314		36 (12)					28 (9)		
**Model 1**										
Conventional tumor budding grade										
Grade 1	57		1 (2)		1.0			1 (2)		1.0
Grade 2	78		6 (8)		3.9; 0.4–42.6 0.252			4 (5)		1.1; 0.09–12.1 0.961
Grade 3	179		29 (16)		8.5; 0.9–74.8 0.058			23 (13)		2.9; 1.1–7.9 0.036
Fibrotic focus										
Absent	210		14 (7)		1.0			9 (4)		1.0
Present	104		22 (21)		2.4; 1.2–5.0 0.017			19 (18)		4.3; 1.8–10.2 < 0.001
Adjuvant therapy										
No	5		2 (40)		1.0			2 (40)		1.0
Yes	309		34 (11)		0.03; 0.007–0.2 < 0.001			26 (8)		0.01; 0.002–0.076 < 0.001
Muscle invasion										
Absent	311		34 (11)		1.0			26 (8)		1.0
Present	3		2 (67)		4.9; 1.1–21.8 0.037			2 (67)		12.3; 2.7–54.0 0.001
Lymph node dissection										
SLN only	208		14 (7)		1.0			10 (5)		1.0
SLN and non-SLN	106		22 (21)		2.1; 1.1–4.2 0.033			18 (17)		2.4; 1.1–5.3 0.028
Histological grade										
Grade 1	66		4 (6)		1.0			2 (3)		1.0
Grade 2	167		12 (7)		0.8; 0.3–2.9 0.764			10 (6)		0.9; 0.2–4.5 0.899
Grade 3	81		20 (25)		2.2; 1.1–4.5 0.031			16 (20)		1.5; 0.3–7.7 0.661
**Model 2**										
Proposed tumor budding grading system										
Grade I	51		0		1.0			0		1.0
Grade II	216		20 (9)		1.0			14 (7)		1.0
Grade III	47		16 (34)		5.1; 2.6–10.1 < 0.001			14 (30)		8.3; 3.7–17.9 < 0.001
Adjuvant therapy										
No	5		2 (40)		1.0			2 (40)		1.0
Yes	309		34 (11)		0.04; 0.008–0.2 < 0.001			26 (8)		0.02; 0.002–0.08 < 0.001
Muscle invasion										
Absent	311		34 (11)		1.0			26 (8)		1.0
Present	3		2 (67)		2.1; 1.1–22.2 0.035			2 (67)		7.3; 1.5–35.1 0.012
Histological grade										
Grade 1	66		4 (6)		1.0			2 (3)		1.0
Grade 2	167		12 (7)		1.2; 0.3–4.2 0.802			10 (6)		1.3; 0.3–6.8 0.723
Grade 3	81		20 (25)		2.4; 1.2–4.9 0.014			16 (20)		2.1; 0.6–10.9 0.376
Tumor necrosis										
Absent	234		19 (8)		1.0			14 (6)		1.0
Present	80		18 (21)		1.6; 0.7–3.5 0.289			14 (18)		2.4; 1.1–5.2 0.034
**Luminal B/HER2-positive and HER2-positive subtypes**										
			Tumor recurrence					Distant-organ metastasis		
			Present		HR; 95% CI *P* value			Present		HR; 95% CI *P* value
	120		17 (14)					15 (13)		
**Model 1**										
Conventional tumor budding grade										
Grade 1	25		0		1.0			0		1.0
Grade 2	30		4 (13)		1.0			4 (13)		1.0
Grade 3	65		13 (20)		1.3; 0.3–5.2 0.764			11 (17)		1.6; 0.4–5.9 0.493
Fibrotic focus										
Absent	76		5 (7)		1.0			5 (7)		1.0
Present	44		12 (27)		3.5; 0.8–15.9 0.113			10 (23)		2.3; 0.5–11.2 0.288
Age (years)										
≤ 39	10		3 (30)		1.0			2 (20)		1.0
> 39	110		14 (13)		0.2; 0.03–0.9 0.032			13 (12)		0.1; 0.02–0.9 0.037
Tumor-infiltrating lymphocytes (%)										
0	1		1 (100)		1.0			1 (100)		1.0
1–19	84		15 (18)		0.1; 0.05–1.9 0.121			13 (16)		0.06; 0.003–1.2 0.063
> 19	35		1 (3)		0.03; 0.009–0.8 0.037			1 (3)		0.009; 0.0002–0.4 0.015
Hormone receptor status										
Negative	54		8 (15)		1.0			8 (15)		1.0
Positive	66		9 (14)		0.3; 0.09–0.9 0.044			7 (11)		0.1; 0.02–0.7 0.022
Perineural invasion										
Absent	104		12 (12)		1.0			10 (10)		1.0
Present	16		5 (31)		3.9; 0.9–16.0 0.059			5 (31)		8.7; 1.5–49.6 0.016
**Model 2**										
Proposed tumor budding grading system										
Grade I	22		0		1.0			0		1.0
Grade II	79		11 (14)		1.0			10 (13)		1.0
Grade III	19		6 (32)		6.6; 1.1–40.6 0.039			5 (26)		9.6; 1.4–64.1 0.020
Age (years)										
≤ 39	10		3 (30)		1.0			2 (20)		1.0
> 39	110		14 (13)		0.1; 0.03–0.7 0.017			13 (12)		0.2; 0.03–1.3 0.092
Tumor-infiltrating lymphocytes (%)										
0	1		1 (100)		1.0			1 (100)		1.0
1–19	84		15 (18)		0.02; 0.0007–0.7 0.031			13 (16)		0.01; 0.0002–0.3 0.010
> 19	35		1 (3)		0.004; 0.0007–0.2 0.005			1 (3)		0.001; 0.00002–0.1 0.001
Hormone receptor status										
Negative	54		8 (15)		1.0			8 (15)		1.0
Positive	66		9 (14)		0.1; 0.02–0.7 0.021			7 (11)		0.06; 0.008–0.4 0.005
Perineural invasion										
Absent	104		12 (12)		1.0			10 (10)		1.0
Present	16		5 (31)		5.5; 1.1–26.8 0.034			5 (31)		10.8; 2.2–51.8 0.003
Invasive tumor size (mm)										
≤ 20	41		4 (10)		1.0			2 (5)		1.0
> 20 to ≤ 50	67		9 (13)		0.6; 0.1–3.1 0.589			9 (13)		2.8; 0.4–20.5 0.325
> 50	12		4 (33)		1.3; 0.1–17.9 0.832			4 (33)		24.4; 1.9–326.1 0.015
**Basal-like subtypes**										
		Cases		TR (%)		HR	95% CI		*P* value	
		87		13 (15)						
**Model 1**										
Conventional tumor budding grade										
Grade 1		26		2 (8)		1.0				
Grade 2		19		2 (11)		1.7	0.2–16.3		0.668	
Grade 3		42		9 (21)		2.3	0.5–11.7		0.316	
Fibrotic focus										
Absent		57		8 (14)		1.0				
Present		30		5 (17)		1.4	0.3–6.2		0.677	
Adjuvant therapy										
No		10		3 (30)		1.0				
Yes		77		10 (13)		0.2	0.04–0.6		0.009	
UICC pN category										
pN0		56		5 (9)		1.0				
pN1		21		3 (14)		0.8	0.2–4.6		0.830	
pN2		5		2 (40)		6.1	1.2–30.8		0.027	
pN3		5		3 (60)		10.6	2.5–44.3		0.001	
**Model 2**										
Proposed tumor budding grading system										
Grade I		22		1 (5)		1.0				
Grade II		56		9 (16)		3.3	0.3–31.6		0.306	
Grade III		9		3 (33)		9.2	0.6–143.2		0.112	
Adjuvant therapy										
No		10		3 (30)		1.0				
Yes		77		10 (13)		0.3	0.03–0.5		0.010	

HR, hazard ratio; CI, confidence interval

Table 6 shows the factors that were significantly associated with tumor recurrence, local recurrence, distant-organ metastasis, and/or tumor-related death according to the patient age; in patients aged ≦39 years, analysis using model 1 failed to demonstrate an association of the CTBG with an increased hazard ratio for tumor recurrence or distant-organ metastasis, while histological grade 3 and radiotherapy were associated with significantly increased hazard ratios for tumor recurrence and distant-organ metastasis. Multivariate analysis using model 2 identified only ProTBGS grade III as being significantly associated with tumor recurrence and distant-organ metastasis (Table 6). In patients aged > 39 years, tumor-infiltrating lymphocytes > 19% was associated with significantly increased hazard ratios for tumor recurrence, distant-organ metastasis, and tumor-related death (Table 6). Multivariate analysis using model 1 identified CTBG grade 3 as well as the presence of an FF and a Ki-67 labeling index of ≧20% as being associated with increased hazard ratios for tumor recurrence and distant-organ metastasis (Table 6). Analysis using model 2 identified ProTBGS grade III and tumor-infiltrating lymphocytes (%) as being associated with significantly increased hazard ratios for tumor recurrence, distant-organ metastasis, and tumor-related death (Table 6).

**Table 6:** Multivariate analyses to identify factors predicting the clinical outcomes in patients with invasive carcinoma of no special type of the breast, according to the age of the patients

**Age, ≤39 years**									
			Tumor recurrence			Distant organ metastasis			
			Present	HR; 95% CI *P*-value		Present		HR; 95% CI *P*-value	
		59	12 (20)			11 (19)			
**Model 1**									
Conventional tumor budding grade									
Grade 1		7	0	1.0		0		1.0	
Grade 2		10	1 (10)	1.0		1 (10)		1.0	
Grade 3		42	11 (26)	3.1; 0.3-32.5 0.347		10 (23)		4.0; 0.4-37.1 0.224	
Fibrotic focus									
Absent		41	4 (10)	1.0		4 (10)		1.0	
Present		18	8 (44)	4.2; 1.2-14.4 0.024		7 (39)		2.6; 0.6-11.5 0.208	
Histological grade									
Grade 1		14	1 (7)	1.0		1 (7)		1.0	
Grade 2		22	3 (14)	0.5; 0.04-6.5 0.609		3 (14)		0.8; 0.2-3.4 0.712	
Grade 3		23	8 (35)	3.9; 1.1-14.5 0.042		7 (30)		4.5; 1.2-16.3 0.024	
Radiotherapy									
No		31	9 (29)	1.0		8 (26)		1.0	
Yes		28	3 (11)	0.1; 0.02-0.8 0.026		3 (11)		0.2; 0.05-0.9 0.044	
HER2 status									
Negative		49	9 (18)	1.0		9 (18)		1.0	
Positive		10	3 (30)	5.6; 1.1-29.3 0.039		2 (20)		0.9; 0.09-9.3 0.913	
Skin invasion									
Absent		54	9 (17)	1.0		8 (15)		1.0	
Present		5	3 (60)	7.7; 0.9-63.8 0.053		3 (60)		13.1; 2.8-63.9 0.002	
**Model 2**									
Proposed tumor budding grading system									
Grade I		7	0	1.0		0		1.0	
Grade II		39	4 (10)	1.0		4 (10)		1.0	
Grade III		13	8 (62)	13.8; 3.5-54.7 <0.001		7 (54)		11.1; 2.6-46.1 0.001	
**Age, >39 years**									
	Cases	No. of patients (%)							
		TR		LR		DOM		TRD	
		+	HR 95%CI P-value	+	HR 95%CI P-value	+	HR 95%CI P-value	+	HR 95%CI P-value
	796	67 (8)		16 (2)		51 (6)		22 (3)	
**Model 1**									
Conventional tumor budding grade									
Grade 1	176	3 (2)	1.0	1 (0.6)	1.0	2 (1)	1.0	1 (0.6)	1.0
Grade 2	198	12 (6)	3.3 0.9-12.2 0.07	3 (1)	3.7 0.4-37.1 0.273	9 (5)	3.2 0.7-16.1 0.151	4 (2)	1.6 0.2-17.0 0.676
Grade 3	422	52 (12)	5.4 1.6-17.7 0.007	12 (3)	5.1 0.6-42.8 0.134	40 (10)	5.7 1.3-25.8 0.023	17 (4)	2.8 0.4-23.5 0.325
Fibrotic focus									
Absent	400	16 (8)	1.0	3 (0.8)	1.0	13 (3)	1.0	6 (2)	1.0
Present	396	51 (13)	2.5 1.5-4.3 0.014	13 (3)	1.4 0.5-4.0 0.547	38 (10)	2.2 1.2-4.4 0.017	16 (4)	2.0 0.7-6.0 0.215
Tumor-infiltrating lymphocytes (%)									
0	6	1 (17)	1.0	0	1.0	1 (17)	1.0	0	1.0
1-19	671	61 (9)	0.4 0.04-3.3 0.375	13 (2)	1.0	48 (7)	0.4 0.04-3.8 0.418	21 (3)	1.0
>19	119	5 (4)	0.2 0.1-0.9 0.031	3 (3)	0.8 0.2-3.1 0.699	2 (2)	0.06 0.01-0.9 0.041	1 (0.8)	0.1 0.01-0.9 0.049
Ki-67 labeling index									
<20	400	16 (4)	1.0	3 (0.8)	1.0	13 (3)	1.0	6 (2)	1.0
>20	396	51 (13)	3.0 1.6-5.3 <0.001	13 (3)	3.7 0.8-1.6 0.081	38 (10)	2.2 1.0-4.9 0.047	16 (4)	0.5 0.1-2.2 0.373
Muscle invasion									
Absent	787	64 (8)	1.0	16 (2)	1.0	48 (6)	1.0	20 (3)	1.0
Present	9	3 (33)	6.3 2.0-21.2 0.002	0	NA	3 (33)	6.9 1.7-27.7 0.007	2 (22)	6.1 0.9-42.1 0.068
Adjuvant therapy									
No	21	4 (19)	1.0	1 (5)	1.0	3 (14)	1.0	0	1.0
Yes	775	63 (8)	0.2 0.06-0.6 0.003	15 (2)	0.7 0.08-3.1 0.787	48 (6)	0.09 0.02-0.4 <0.001	22 (3)	NA
Hormone receptor status									
Negative	132	20 (15)	1.0	6 (5)	1.0	14 (11)	1.0	11 (8)	1.0
Positive	664	47 (7)	0.5 0.3-0.9 0.011	10 (2)	0.4 0.1-1.4 0.128	37 (6)	0.5 0.2-1.2 0.136	11 (2)	0.3 0.1-0.8 0.016
UICC pN category									
pN0	573	31 (3)	1.0	9 (2)	1.0	22 (4)	1.0	7 (1)	1.0
pN1	148	20 (14)	1.8 0.7-4.7 0.256	4 (3)	0.9 0.3-3.0 0.881	16 (11)	2.0 0.7-6.1 0.227	7 (5)	3.8 1.3-11.1 0.016
pN2	49	9 (18)	2.0 0.6-6.5 0.267	0	0.9 0.3-3.0 0.881	9 (18)	2.8 0.7-11.2 0.141	5 (10)	6.2 1.8-21.6 0.004
pN3	26	7 (27)	2.6 0.8-9.3 0.128	3 (12)	5.3 1.5-19.0 0.011	4 (15)	2.24 0.5-10.4 0.302	3 (12)	9.7 2.3-41.1 0.002
Histological grade									
Grade 1	250	9 (13)	1.0	2 (0.8)	1.0	7 (3)	1.0	2 (0.8)	1.0
Grade 2	330	22 (7)	0.9 0.4-2.0 0.798	4 (1)	0.8 0.2-4.8 0.818	18 (6)	0.9 0.4-2.3 0.852	3 (1)	0.3 0.05-2.4 0.282
Grade 3	216	36 (17)	1.1 0.4-2.7 0.902	10 (5)	3.8 1.4-10.8 0.011	26 (12)	0.9 0.3-2.5 0.773	17 (8)	4.6 1.5-14.0 0.007
Skin invasion									
Absent	717	53 (7)	1.0	12 (2)	1.0	41 (6)	1.0	15 (2)	1.0
Present	79	17 (18)	2.5 1.4-4.6 0.004	4 (5)	2.7 0.6-11.3 0.174	10 (13)	1.4 0.6-3.3 0.440	7 (9)	1.7 0.5-5.7 0.406
Radiotherapy									
No	438	45 (10)	1.0	12 (3)	1.0	33 (8)	1.0	15 (3)	1.0
Yes	358	22 (6)	0.6 0.3-1.0 0.050	4 (1)	0.4 0.1-1.3 0.126	18 (5)	0.6 0.3-1.2 0.160	7 (2)	0.3 0.1-0.9 0.030
Perineural invasion									
Absent	652	49 (8)	1.0	13 (2)	1.0	36 (6)	1.0	10 (2)	1.0
Present	144	18 (13)	1.1 0.6-2.2 0.707	3 (2)	0.6 0.2-2.2 0.438	15 (10)	1.3 0.6-2.7 0.478	12 (8)	7.4 2.9-19.0 <0.001
**Model 2**									
Proposed tumor budding grading system									
Grade I	157	1 (0.6)	1.0	0	1.0	1 (0.6)	1.0	1 (0.6)	1.0
Grade II	542	42 (8)	12.1 1.6-92.8 0.016	12 (2)	1.0	30 (6)	4.2 1.1-69.3 0.040	11 (2)	1.2 0.1-10.2 0.851
Grade III	97	24 (25)	31.4 3.9-257.5 0.001	4 (4)	2.1 0.6-7.1 0.247	20 (21)	26.7 3.1-234 0.003	10 (10)	4.2 1.6-11.1 0.003
Tumor-infiltrating lymphocytes (%)									
0	6	1 (17)	1.0	0	1.0	1 (17)	1.0	0	1.0
1-19	671	61 (9)	0.2 0.02-1.8 0.150	13 (2)	1.0	48 (7)	0.2 0.02-1.9 0.162	21 (3)	1.0
>19	119	5 (4)	0.06 0.001-0.7 0.022	3 (3)	0.8 0.2-3.2 0.736	2 (2)	0.03 0.01-0.4 0.009	1 (0.8)	0.1 0.01-0.8 0.034
Adjuvant therapy									
No	21	4 (19)	1.0	1 (5)	1.0	3 (14)	1.0	0	1.0
Yes	775	63 (8)	0.2 0.05-0.5 0.003	15 (2)	0.5 0.05-4.5 0.526	48 (6)	0.1 0.03-0.5 0.001	22 (3)	NA
Hormone receptor status									
Negative	132	20 (15)	1.0	6 (5)	1.0	14 (11)	1.0	11 (8)	1.0
Positive	664	47 (7)	0.5 0.2-0.9 0.026	10 (2)	0.4 0.1-1.4 0.150	37 (6)	0.5 0.9-1.1 0.065	11 (2)	0.08 0.02-0.2 <0.001
Ki-67 labeling index									
<20	400	16 (4)	1.0	3 (0.8)	1.0	13 (3)	1.0	6 (2)	1.0
>20	396	51 (13)	2.4 1.2-4.7 0.015	13 (3)	3.1 0.7-12.8 0.125	38 (10)	2.1 0.9-4.6 0.063	16 (4)	0.5 0.1-2.0 0.302
Muscle invasion									
Absent	787	64 (8)	1.0	16 (2)	1.0	48 (6)	1.0	20 (3)	1.0
Present	9	3 (33)	4.9 1.3-17.9 0.016	0	NA	3 (33)	7.6 1.9-30.0 0.004	2 (22)	9.1 1.7-49.2 0.010
Histological grade									
Grade 1	250	9 (13)	1.0	2 (0.8)	1.0	7 (3)	1.0	2 (0.8)	1.0
Grade 2	330	22 (7)	0.9 0.4-2.1 0.872	4 (1)	0.9 0.2-5.6 0.968	18 (6)	0.9 0.4-2.4 0.927	3 (1)	0.4 0.06-2.7 0.343
Grade 3	216	36 (17)	1.1 0.4-2.8 0.777	10 (5)	3.2 1.0-9.9 0.043	26 (12)	0.9 0.3-2.7 0.896	17 (8)	4.0 1.3-11.6 0.013
UICC pN category									
pN0	573	31 (3)	1.0	9 (2)	1.0	22 (4)	1.0	7 (1)	1.0
pN1	148	20 (14)	1.5 0.5-3.8 0.462	4 (3)	1.1 0.3-3.6 0.871	16 (11)	1.5 0.5-4.8 0.451	7 (5)	1.7 0.3-9.6 0.560
pN2	49	9 (18)	1.7 0.5-3.8 0.413	0	1.1 0.3-3.6 0.871	9 (18)	2.2 0.6-8.8 0.247	5 (10)	2.9 0.4-23.3 0.313
pN3	26	7 (27)	1.8 0.5-6.4 0.395	3 (12)	7.1 2.5-25.4 0.003	4 (15)	1.4 0.3-6.8 0.689	3 (12)	1.9 0.2-18.1 0.580
Perineural invasion									
Absent	652	49 (8)	1.0	13 (2)	1.0	36 (6)	1.0	10 (2)	1.0
Present	144	18 (13)	1.1 0.6-2.2 0.707	3 (2)	0.6 0.2-2.6 0.512	15 (10)	1.3 0.6-2.7 0.478	12 (8)	4.3 1.7-11.1 0.002
Blood vessel invasion									
Absent	525	33 (6)	1.0	8 (2)	1.0	26 (5)	1.0	8 (2)	1.0
Present	271	34 (13)	1.4 0.8-2.5 0.244	8 (3)	1.8 0.6-5.4 0.332	26 (10)	1.4 0.7-2.7 0.379	14 (5)	3.6 1.2-10.3 0.018

HR, hazard ratio; CI, confidence interval; TR, tumor recurrence; LR, local recurrence; DOM, distant-organ metastasis; TRD, tumor-related death; + , present

Supplementary Table 3 shows the factors that were found by multivariate analyses as being significantly associated with tumor recurrence, local recurrence, distant-organ metastasis, and/or overall survival, according to the Ki-67 labeling index. In cases with a Ki-67 labeling index ≤ 20%, analysis using model 1 failed to demonstrate any significant association of the CTBG with increased hazard ratios for tumor recurrence or distant-organ metastasis; on the other hand, presence of FF, invasive tumor size > 50 mm, and age ≦39 years were associated with significantly increased hazard ratios for tumor recurrence and distant-organ metastasis (Supplementary Table 3). Multivariate analysis using model 2 identified ProTBGS grade III and invasive tumor size > 50 mm as being associated with increased hazard ratio for tumor recurrence and distant-organ metastasis (Supplementary Table 2). In cases with a Ki-67 labeling index of > 20%, CTBG grade 3 only significantly increased hazard ratio for tumor recurrence, while analysis using model 2 identified ProTBGS grade III as well as the presence of muscle invasion as being associated with increased hazard ratios for tumor recurrence, distant-organ metastasis, and tumor-related death (Supplementary Table 3).

Supplementary Table 4 shows the factors that were found by multivariate analyses as being significantly associated with tumor recurrence, local recurrence, distant-organ metastasis, and/or tumor-related death, according to histological grade. Multivariate analysis using model 1 failed to demonstrate any significant association of CTBG with tumor recurrence, local recurrence, distant-organ metastasis, or tumor-related death in histological grade 1, 2, and 3 group. Multivariate analysis using model 2 identified ProTBGS grade III as showing no significant association with tumor recurrence in cases with histological grade 1 tumors. In cases with histological grade 2 tumors, analysis using model 2 identified ProTBGS grade III as well as presence of muscle invasion and invasive tumor size > 50 mm as being associated with increased hazard ratios for tumor recurrence and distant-organ metastasis. In cases with histological grade 3 tumors, analysis using model 2 identified ProTBGS grade III as being associated with increased hazard ratio only for tumor-related death (Supplementary Table 4).

## Discussion

ProTBGS, which additionally incorporated the TBG in an FF, where present, as compared to CTBG, was clearly demonstrated to show superior ability for accurately predicting the outcomes in patients with ICNST of the breast (Table 7). As an FF is composed of cancer-associated fibroblasts, thus, ProTBGS also incorporates the aspect of tumor cell–stromal cell interaction within the FF [32–34], which have been reported as playing an important role in accelerating tumor progression in carcinomas of various organs [35–37]. We and others have previously reported that the FF is a very important prognostic parameter in patients with ICNST of the breast [8–18], and recently, tumor cell–stromal cell interactions have also been identified as playing important roles in colorectal carcinoma and pancreatic carcinoma [37–39]. In addition, in the present study, assessment by the ProTBGS was demonstrated to show superior power to that by the presence/absence of an FF alone for accurately predicting the outcome in patients with ICNST (Table 7); this probably indicates that assessment according to ProTBGS is superior to that by the presence/absence of an FF alone for accurate assessment of the characteristics of the tumor cells and tumor–stromal cell interaction in patients with ICNST. Thus, incorporation of tumor cell–stromal cell interactions in the evaluation is probably the reason why the outcome-predictive power of ProTBGS was found to be superior. Table 7 clearly demonstrates that the ProTBGS grade III was highly powerful for accurately predicting the clinical outcomes in patients with ICNSTs. Salhia et al. reported that intratumor budding (ITB) as well as peripheral tumor budding (PTB; equal to CTBG) were important prognostic factors in patients with invasive breast carcinoma, and that cases should be examined for both ITB and PTB [6]; we also investigated the prognostic power of ITB as well as PTB, and concluded that both tumor budding had almost similar prognostic power each other (Supplementary Table 5). Although ITB is probably an important prognostic indicator as well as PTB, ITB cannot reflect degree of tumor–stromal cell interaction in ICNST; in contrast, TBG in the FF can more accurately reflect degree of tumor–stromal cell interaction in ICNST than ITB. Therefore, we conducted to make a more powerful TB grading system than CTBG using TBG in the FF, and the results of the present study clearly revealed that ProTBGS was a superior TBG system to CTBG in patients with ICNST. Therefore, we concluded that the ProTBGS is the most reliable histological grading system for accurately predicting the outcomes in patients with ICNSTs of the breast. In the case of colorectal carcinoma, tumor budding is known as an independent predictor of survival in UICC stage II colorectal cancer patients [26]; ProTBGS grade III clearly demonstrated an excellent outcome-predictive power in patients with ICNST of the breast, independent of UICC pTNM stage, which strongly suggests that the incorporation of the tumor cell–stromal cell interactions enhance the outcome-predictive power of ProTBGS. Therefore, evaluation of the tumor budding grade in fibrotic tumor stroma, corresponding to the TBG in an FF in ICNSTs, in cases of colorectal cancer, pancreatic cancer, and other cancers may be very useful to analyze tumor cell nests and interactions of the tumor cells–stroma cells surrounding the tumor cell nests in colorectal cancer and other cancers [40–44].

Histological grade is the histological predictor of the outcome in patients with ICNST of the breast that is accepted worldwide [45]; the present study clearly demonstrated that assessment by the ProTBGS was superior to that of the histological grade for predicting the outcomes of patients with ICNST of the breast, and that the ProTBGS is also useful to accurately predict the outcomes of patients with ICNST of the breast of different histological grade. Thus, ProTBGS showed the best power among all histological parameters for predicting the outcomes in patients with ICNST; furthermore, use of the ProTBGS even allowed identification of patients with high-grade malignancy separately among patients classified as histological grade 1, 2, and 3. In addition, since ProTBGS is also a very useful outcome predictor in patients with ICNSTs independent of intrinsic subtype, patient age, or Ki-67 labeling index, we can conclude that ProTBGS is a very useful outcome predictor in patients with ICNNSTs, independent of the biological characteristics of the tumor/patients. Thus, we encourage pathologists to report ProTBGS in the routine pathological report of surgical material of ICNSTs of the breast, and in biopsy material [46], we suggest that the pathologist examine the TBG in the FF (resembling a fibrotic scar region within the fibrotic tumor stroma), if present within the ICNST, in addition to examining the CTB.

In conclusion, this study demonstrated that use of the ProTBGS is superior to that of CTBG, assessment of the histological grade, and assessment of the presence/absence of an FF for accurate prediction of the outcomes in patients with ICNST; therefore, ProTBGS is probably the most reliable histological grading system at present for predicting the prognosis in patients with ICNST of the breast. ProTBGS additionally incorporates assessment of TB in an FF, as compared to CTBG; this strongly suggests that the integrated actions of tumor-stromal fibroblasts forming an FF and tumor budding cells in the FF probably heighten the malignant potential of ICNSTs with an FF. Thus, factors that are produced by tumor cell–tumor stromal cell interactions should be investigated for the development of targeted therapies for patients with ICNST; ProTBGS may be very useful for histological selection of patients with ICNST for therapy targeted at tumor–stromal cell–tumor cell interactions.

## Supplementary Information

Below is the link to the electronic supplementary material.

Supplementary file1 (PDF 206 KB)
