# Correction to: Tumor budding and fibrotic focus—proposed grading system for tumor budding in invasive carcinoma no special type of the breast

**DOI:** 10.1007/s00428-022-03486-2

**Published:** 2022-12-29

**Authors:** Miyuki Hiratsuka, Takahiro Hasebe, Yuki Ichinose, Ayaka Sakakibara, Akihiro Fujimoto, Noriko Wakui, Satomi Shibasaki, Masataka Hirasaki, Masanori Yasuda, Akemi Nukui, Hiroko Shimada, Hideki Yokogawa, Kazuo Matsuura, Takashi Hojo, Akihiko Osaki, Toshiaki Saeki

**Affiliations:** 1 Department of Breast Oncology, Saitama Medical University International Medical Center, 1397-1, Yamane, Hidaka City, Saitama 350-1298 Japan; 2 Community Health Science Center, Saitama Medical University, 29, Morohongou, Moroyama Town, Iruma district, Saitama 350-0495 Japan; 3 Department of Clinical Cancer Genomics, Saitama Medical University International Medical Center, 1397-1, Yamane, Hidaka City, Saitama 350-1298 Japan; 4 Department of Pathology, Saitama Medical University International Medical Center, 1397-1, Yamane, Hidaka City, Saitama 350-1298 Japan

**Correction to: Virchows Archiv (2022) 481:161-190**

10.1007/s00428-022-03337-0

The data under Materials and methods, section “Patients and histological examinations” in the original published version of the above article has been corrected.

Original sentence says, "The subjects of this study were 855 consecutive patients with ICNST of the breast who had undergone surgical treatment without prior neoadjuvant therapy at the Saitama Medical University International Medical Center between **January 2007 and December 2015**." was corrected to "The subjects of this study were 855 consecutive patients with ICNST of the breast who had undergone surgical treatment without prior neoadjuvant therapy at the Saitama Medical University International Medical Center between **April 2007 and December 2015**." [Bold emphasis used to highlight the corrected area] 

The original article has been corrected.

