# Supplementary material for: Tumor budding and fibrotic focus—proposed grading system for tumor budding in invasive carcinoma no special type of the breast
Source: Virchows Arch. 2022 Jun 13;481(2):161–90. doi: 10.1007/s00428-022-03337-0 (PMC9343319; doi:10.1007/s00428-022-03337-0)
Supplement: Supplementary file 1 — Supplementary file1 (PDF 206 KB) [file 428_2022_3337_MOESM1_ESM.pdf]

Supplementary Table 1 Clinicopathological factors and outcomes (univariate analyses)

	Cases	TR (%)	LR (%)	DOM (%)	TRD (%)
	855	79	17	62	26
Age (years)					
≤39	59	12 (20)	1 (2)	11 (19)	4 (7)
>39	796	67 (8)	16 (2)	51 (6)	22 (3)
p-values		0.005	0.851	0.001	0.149
Adjuvant therapy					
No	22	5 (23)	1 (5)	4 (18)	1 (5)
Yes	833	74 (9)	16 (2)	58 (7)	25 (3)
p-values		0.001	0.221	0.003	0.294
Radiotherapy					
No	469	54 (12)	13 (3)	41 (9)	18 (4)
Yes	386	25 (7)	4 (1)	21 (5)	8 (2)
p-values		0.013	0.076	0.065	0.154
Hormone receptor status					
Negative	141	21 (15)	6 (4)	15 (11)	12 (9)
Positive	714	58 (8)	11 (2)	47 (7)	14 (2)
p-values		0.011	0.040	0.074	<0.001
HER2 status					
Negative	732	62 (9)	15 (2)	47 (6)	19 (3)
Positive	123	17 (14)	2 (2)	15 (12)	7 (6)
p-values		0.010	0.707	0.039	0.116
Ki-67 labeling index					
<20	420	20 (5)	3 (0.7)	17 (4)	7 (2)
≥20	435	59 (14)	14 (3)	45 (10)	19 (4)
p-values		<0.001	0.017	<0.001	0.027
Intrinsic subtype					
Luminal A	334	13 (4)	1 (0.3)	12 (4)	3 (1)
Luminal B (HER2-)	314	36 (12)	8 (3)	28 (9)	9 (3)
Luminal B (HER2+)	66	9 (14)	2 (3)	7 (11)	2 (3)
HER2	54	8 (15)	0	8 (15)	5 (9)
Basal-like	87	13 (15)	6 (7)	7 (8)	7 (8)
p-values		<0.001	0.002	0.011	<0.001
Adipose tissue invasion					
Absent	55	3 (5)	1 (2)	2 (4)	0
Present	800	76 (10)	16 (2)	60 (8)	26

p-values		0.270	0.904	0.078	NA
Blood vessel invasion					
Absent	570	42 (7)	8 (1)	34 (6)	11 (2)
Present	285	37 (13)	9 (3)	28 (10)	15 (5)
p-values		0.002	0.071	0.012	0.004
Fibrotic focus					
Absent	609	31 (5)	8 (1)	23 (4)	7 (1)
Present	246	48 (20)	9 (4)	39 (16)	19 (8)
p-values		<0.001	0.026	<0.001	<0.001
Histological grade					
Grade 1	264	10 (4)	2 (0.8)	8 (3)	2 (1)
Grade 2	352	25 (7)	4 (1)	21 (6)	4 (1)
Grade 3	239	44 (18)	11 (5)	33 (14)	20 (8)
p-values		<0.001	0.432	<0.001	<0.001
Tumor-infiltrating lymphocytes (%)					
0	6	1 (17)	0	1 (17)	0
1-19	725	72 (10)	14 (2)	58 (8)	25 (4)
>19	124	6 (5)	3 (2)	3 (2)	1 (0.8)
p-values		0.063	0.689	0.020	0.164
Invasive tumor size (mm)					
≤20	326	13 (4)	5 (2)	8 (3)	1 (0.3)
>20 - ≤50	483	52 (11)	9 (2)	43 (9)	18 (4)
>50	46	14 (30)	3 (7)	11 (24)	7 (15)
p-values		<0.001	0.117	<0.001	<0.001
Lymph vessel invasion					
Absent	570	40 (7)	8 (1)	32 (6)	14 (3)
Present	285	39 (14)	9 (3)	30 (11)	12 (4)
p-values		0.007	0.106	0.028	0.256
Muscle invasion					
Absent	845	75 (9)	17 (2)	58 (7)	23 (3)
Present	10	4 (40)	0	4 (40)	3 (30)
p-values		<0.001	NA	<0.001	<0.001
Perineural invasion					
Absent	702	58 (8)	14 (2)	44 (6)	13 (2)
Present	153	21 (14)	3 (2)	18 (12)	13 (9)
p-values		0.014	0.933	0.006	<0.001
Skin invasion					
Absent	771	62 (8)	14 (2)	44 (6)	17 (2)

Present	85	17 (20)	3 (2)	18 (12)	9 (11)
p-values		<0.001	0.933	0.006	<0.001
Tumor necrosis					
Absent	625	42 (7)	10 (2)	32 (5)	10 (2)
Present	230	37 (16)	7 (3)	30 (13)	16 (7)
p-values		<0.001	0.840	0.292	<0.001
Lymph node dissection					
SLN only	579	32 (6)	9 (2)	23 (4)	8 (1)
SLN and non-SLN	276	47 (17)	8 (3)	39 (14)	18 (7)
p-values		<0.001	0.193	<0.001	<0.001
UICC pN category					
pN0	610	35 (6)	10 (2)	25 (4)	9 (2)
pN1	163	25 (15)	4 (3)	21 (13)	7 (4)
pN2	54	10 (19)	0	10 (19)	6 (11)
pN3	28	9 (35)	3 (11)	6 (21)	4 (14)
p-values		<0.001	0.041	<0.001	<0.001
UICC pTNM stage classification					
IA/B	286	12 (4)	4 (1)	8 (3)	1 (0.4)
IIA	319	21 (7)	6 (2)	15 (5)	6 (2)
IIB	116	18 (16)	2 (2)	16 (14)	6 (5)
IIIA	42	6 (14)	1 (2)	5 (12)	1 (2)
IIIB	67	14 (21)	2 (3)	12 (18)	9 (13)
IIIC	25	8 (32)	2 (8)	6 (24)	3 (12)
p-values		<0.001	0.055	<0.001	<0.001

TR, tumor recurrence; LR, local recurrence; DOM, distant-organ metastasis; TRD, tumor-related death; NA, not available; SLN, sentinel lymph node; HER2-, HER2-negative; HER2+, HER2-positive

Supplementary Table 2 In situ HER hybridization and HER2 immunohistochemistry assessment according to the 2018 ASCO CAP recommendation

Group	Biology	HER2/CEP 17	Mean HER2 copy Number	2018 ASCO CAP recommendation
1	Classic HER2 amplified	≥2.0	≥4.0	Positive
2	Monosomy 17	≥2.0	<4.0	Positive: IHC 3+
		≥2.0	<4.0	Negative: IHC 1+ or 2+
3	Co-amplification, previously polysomy 17	<2.0	≥6.0	Positive: IHC 2+ or 3+
		<2.0	≥6.0	Negative: IHC 1+
4	Borderline	<2.0	≥4.0 and <6.0	Positive: IHC 3+
		<2.0	≥4.0 and <6.0	Negative: IHC 1+ or 2+
5	Classic HER2 nonamplified	<2.0	<4.0	Negative

HER, human epidermal growth factor receptor-2; IHC, immunohistochemistry

Supplementary Table 3 Multivariate analyses to identify factors predicting the clinical outcomes in patients with invasive carcinoma of no special type of the breast, according to the Ki-67 labeling index

**Ki-67 labeling index, <20%**					
		Tumor recurrence		Distant organ metastasis	
		Present	HR; 95% CI<br>*P*-value	Present	HR; 95% CI<br>*P*-value
	420	20 (5)		17 (4)	
			**Model 1**		
Conventional tumor budding grade					
Grade 1	96	0	1.0	0	1.0
Grade 2	103	4 (4)	1.0	3 (3)	1.0
Grade 3	221	16 (7)	1.1; 0.3-3.8	14 (6)	1.6; 0.4-7.0
			0.942		0.527
Fibrotic focus					
Absent	329	6 (2)	1.0	5 (2)	1.0
Present	91	14 (15)	6.5; 1.6-19.3	12 (13)	8.2; 2.8-23.7
			<0.001		<0.001
Invasive tumor size (mm)					
≤20	189	2 (1)	1.0	2 (1)	1.0
>20 - ≤50	216	13 (6)	4.4; 0.8-23.0	11 (5)	2.7; 0.5-14.2
			0.082		0.248
>50	15	5 (33)	46.1; 5.6-367.9	4 (27)	5.1; 1.6-17.0
			<0.001		0.008
Age (year)					
≤39	20	4 (20)	1.0	4 (20)	1.0
>39	400	16 (4)	0.2; 0.04-0.8	13 (3)	0.2; 0.04-0.6
			0.020		0.005
			**Model 2**		
Proposed tumor budding grading system					
Grade I	88	0	1.0	0	1.0
Grade II	288	11 (4)	1.0	9 (3)	1.0
Grade III	44	9 (21)	4.0; 1.1-14.4	8 (18)	6.9; 2.5-19.1
			0.032		<0.001
Invasive tumor size (mm)					
≤20	189	2 (1)	1.0	2 (1)	1.0
>20 - ≤50	216	13 (6)	5.4; 0.9-30.1	11 (5)	5.0; 0.8-31.3

			0.056		0.087
>50	15	5 (33)	42.2; 5.2-316.7<br><0.001	4 (27)	28.3; 2.6-268.8<br>0.004
Age (year)					
≤39	20	4 (20)	1.0	4 (20)	1.0
>39	400	16 (4)	0.2; 0.05-1.1<br>0.052	13 (3)	0.2; 0.06-0.6<br>0.007

Ki-67 labeling index, ≥20%

	Cases	No. of patients (%)							
		TR		LR		DOM		TRD	
		+	HR<br>95%CI<br>P-value	+	HR<br>95%CI<br>P-value	+	HR<br>95%CI<br>P-value	+	HR<br>95%CI<br>P-value
	435	59<br>(14)		14<br>(3)		45<br>(10)		19<br>(4)	
				**Model 1**					
Conventional tumor budding grade									
Grade 1	87	3<br>(3)	1.0	1<br>(1)	1.0	2<br>(2)	1.0	1<br>(1)	1.0
Grade 2	105	9<br>(9)	2.2<br>0.6-8.4<br>0.259	2<br>(2)	2.3<br>0.2-26.8<br>0.516	7<br>(7)	2.4<br>0.5-12.5<br>0.292	3<br>(3)	0.7<br>0.06-7.9<br>0.774
Grade 3	243	47<br>(19)	3.7<br>1.1-12.2<br>0.039	11<br>(5)	3.7<br>0.5-30.2<br>0.222	36<br>(15)	3.8<br>0.8-17.3<br>0.083	15<br>(6)	2.1<br>0.2-17.2<br>0.498
Fibrotic focus									
Absent	280	25<br>(9)	1.0	7<br>(3)	1.0	18<br>(6)	1.0	5<br>(2)	1.0
Present	155	34<br>(22)	1.5<br>0.8-2.7<br>0.203	7<br>(5)	1.1<br>0.3-3.7<br>0.845	27<br>(17)	1.5<br>0.7-3.2<br>0.237	14<br>(9)	1.9<br>0.6-6.5<br>0.296
Adjuvant therapy									
No	12	4<br>(33)	1.0	1<br>(8)	1.0	3<br>(25)	1.0	0	1.0
Yes	423	55<br>(13)	0.1<br>0.03-0.4<br><0.001	13<br>(3)	0.3<br>0.03-2.3<br>0.222	42<br>(10)	0.08<br>0.002-0.4<br>0.001	19<br>(5)	NA
Radiotherapy									

No	257	42<br>(16)	1.0	12<br>(5)	1.0	30<br>(12)	1.0	13<br>(5)	1.0
Yes	178	17<br>(10)	0.5<br>0.3-0.9<br>0.020	2<br>(1)	0.2<br>0.04-0.9<br>0.034	15<br>(8)	0.5<br>0.3-1.1<br>0.087	6<br>(3)	0.5<br>0.1-1.6<br>0.228
Tumor-infiltrating lymphocytes (%)									
0	1	1<br>(100)	1.0	0	1.0	1<br>(100)	0	0	1.0
1-19	346	54<br>(16)	1.0	12<br>(4)	1.0	42<br>(12)	0.2<br>0.02-2.8<br>0.249	19<br>(6)	1.0
>19	88	4<br>(5)	0.3<br>0.09-0.9<br>0.026	2<br>(2)	0.8<br>0.2-4.1<br>0.772	2<br>(2)	0.05<br>0.001-0.8<br>0.033	0	NA
Muscle invasion									
Absent	431	56<br>(13)	1.0	14<br>(3)	1.0	42<br>(10)	1.0	16<br>(4)	1.0
Present	4	3<br>(75)	4.2<br>1.1-15.8<br>0.035	0	NA	3<br>(75)	3.7<br>0.9-15.3<br>0.072	3<br>(75)	22.8<br>3.1-167.2<br>0.002
UICC pN category									
pN0	296	27<br>(9)	1.0	8<br>(3)	1.0	19<br>(6)	1.0	6<br>(2)	1.0
pN1	84	18<br>(21)	1.9<br>0.6-5.7<br>0.246	4<br>(5)	1.4<br>0.4-5.1<br>0.569	14<br>(17)	2.1<br>0.6-7.7<br>0.250	6<br>(7)	2.6<br>0.8-9.2<br>0.129
pN2	36	8<br>(22)	1.7<br>0.4-6.5<br>0.449	0	1.4<br>0.4-5.1<br>0.569	8<br>(22)	2.2<br>0.5-10.7<br>0.317	5<br>(14)	2.7<br>0.5-13.9<br>0.243
pN3	19	6<br>(32)	3.5<br>0.9-14.2<br>0.078	2<br>(11)	6.2<br>1.3-28.5<br>0.019	4<br>(21)	3.4<br>0.6-18.7<br>0.153	2<br>(11)	4.8<br>0.7-32.2<br>0.105
Perineural invasion									
Absent	360	45<br>(13)	1.0	13<br>(4)	1.0	32<br>(9)	1.0	8<br>(2)	1.0
Present	75	14<br>(19)	1.2<br>0.6-2.4<br>0.688	1<br>(1)	0.2<br>0.02-1.6<br>0.133	13<br>(17)	1.9<br>0.8-4.2<br>0.121	11<br>(15)	6.7<br>2.0-22.1<br>0.002

Hormone receptor status									
Negative	118	18<br>(15)	1.0	4<br>(3)	1.0	14<br>(12)	1.0	9<br>(8)	1.0
Positive	317	41<br>(13)	0.8<br>0.4-1.5<br>0.437	10<br>(3)	0.9<br>0.2-4.1<br>0.944	31<br>(10)	0.7<br>0.4-1.5<br>0.346	10<br>(3)	0.2<br>0.04-0.7<br>0.014
Histological grade									
Grade 1	54	4<br>(7)	1.0	2<br>(4)	1.0	2<br>(4)	1.0	0	1.0
Grade 2	175	15<br>(9)	0.8<br>0.3-2.5<br>0.667	2<br>(1)	0.3<br>0.04-2.0<br>0.196	13<br>(7)	1.2<br>0.3-5.7<br>0.806	2<br>(1)	1.0
Grade 3	206	40<br>(19)	1.4<br>0.4-4.5<br>0.560	10<br>(5)	3.1<br>0.9-10.9<br>0.074	30<br>(15)	1.6<br>0.3-7.8<br>0.543	17<br>(8)	9.5<br>1.4-65.4<br>0.021
**Model 2**									
Proposed tumor budding grading system									
Grade I	76	1<br>(1)	1.0	0	1.0	1<br>(1)	1.0	1<br>(1)	1.0
Grade II	293	35<br>(12)	6.9<br>0.9-52.8<br>0.060	10<br>(3)	1.0	25<br>(9)	5.0<br>0.6-39.2<br>0.126	8<br>(3)	0.8<br>0.1-7.2<br>0.865
Grade III	66	23<br>(35)	17.1<br>2.0-123.9<br>0.008	4<br>(6)	2.2<br>0.6-7.9<br>0.224	19<br>(29)	14.0<br>1.7-118.7<br>0.016	10<br>(15)	4.3<br>1.3-15.4<br>0.019
Adjuvant therapy									
No	12	4<br>(33)	1.0	1<br>(8)	1.0	3<br>(25)	1.0	0	1.0
Yes	423	55<br>(13)	0.1<br>0.04-0.4<br>0.001	13<br>(3)	0.2<br>0.03-2.3<br>0.200	42<br>(10)	0.1<br>0.02-0.4<br>0.002	19<br>(5)	NA
Tumor-infiltrating lymphocytes (%)									
0	1	1<br>(100)	1.0	0	1.0	1<br>(100)	1.0	0	1.0
1-19	346	54<br>(16)	1.0	12<br>(4)	1.0	42<br>(12)	0.1<br>0.001-1.5<br>0.101	19<br>(6)	1.0
>19	88	4	0.3	2	0.6	2	0.02	0	NA

		(5)	0.09-0.9<br>0.026	(2)	0.1-3.1<br>0.579	(2)	0.001-0.4<br>0.010		
Muscle invasion									
Absent	431	56<br>(13)	1.0	14<br>(3)	1.0	42<br>(10)	1.0	16<br>(4)	1.0
Present	4	3<br>(75)	4.7<br>1.2-18.2<br>0.024	0	NA	3<br>(75)	4.4<br>1.0-19.1<br>0.045	3<br>(75)	40.2<br>4.7-349.3<br><0.001
Radiotherapy									
No	257	42<br>(16)	1.0	12<br>(5)	1.0	30<br>(12)	1.0	13<br>(5)	1.0
Yes	178	17<br>(10)	0.5<br>0.3-0.9<br>0.029	2<br>(1)	0.2<br>0.05-1.1<br>0.053	15<br>(8)	0.6<br>0.3-1.1<br>0.107	6<br>(3)	0.6<br>0.2-1.9<br>0.388
Perineural invasion									
Absent	360	45<br>(13)	1.0	13<br>(4)	1.0	32<br>(9)	1.0	8<br>(2)	1.0
Present	75	14<br>(19)	1.0<br>0.5-2.1<br>0.955	1<br>(1)	0.2<br>0.02-1.6<br>0.125	13<br>(17)	1.6<br>0.7-3.7<br>0.256	11<br>(15)	6.1<br>1.7-21.4<br>0.005
Hormone receptor status									
Negative	118	18<br>(15)	1.0	4<br>(3)	1.0	14<br>(12)	1.0	9<br>(8)	1.0
Positive	317	41<br>(13)	0.7<br>0.3-1.3<br>0.243	10<br>(3)	0.9<br>0.2-4.0<br>0.854	31<br>(10)	0.5<br>0.2-1.3<br>0.149	10<br>(3)	0.1<br>0.03-0.6<br>0.007
Histological grade									
Grade 1	54	4<br>(7)	1.0	2<br>(4)	1.0	2<br>(4)	1.0	0	1.0
Grade 2	175	15<br>(9)	0.8<br>0.2-2.4<br>0.622	2<br>(1)	0.3<br>0.04-2.2<br>0.240	13<br>(7)	1.1<br>0.3-5.2<br>0.867	2<br>(1)	1.0
Grade 3	206	40<br>(19)	1.3<br>0.4-4.1<br>0.628	10<br>(5)	3.7<br>1.0-13.8<br>0.045	30<br>(15)	1.5<br>0.3-7.0<br>0.632	17<br>(8)	9.6<br>1.5-64.2<br>0.020

HR, hazard ratio; CI, confidence interval; TR, tumor recurrence; LR, local recurrence; DOM, distant-organ metastasis; TRD, tumor-related death; +, present

Supplementary Table 4 Multivariate analyses to identify predictors of the clinical outcomes in patients with invasive carcinoma of no special type of the breast according to the tumor histological grade

**Histological grade 1**					
		**Tumor recurrence**			
	Cases	TRR (%)	HR	95% CI	p-value
	264	10 (4)			
			**Model 1**		
Conventional tumor budding grade					
Grade 1	94	0	1.0		
Grade 2	71	2 (3)	1.0		
Grade 3	99	8 (8)	2.8	0.4-17.5	0.278
Fibrotic focus					
Absent	231	4 (2)	1.0		
Present	33	6 (18)	5.3	1.3-22.0	0.024
			**Model 2**		
Proposed tumor budding grading system					
Grade I	90	0	1.0		
Grade II	160	6 (4)	1.0		
Grade III	14	4 (19)	3.4	0.7-16.4	0.136
Blood vessel invasion					
Absent	184	3 (2)	1.0		
Present	80	7 (9)	6.0	1.5-23.6	0.010
Radiotherapy					
No	133	8 (6)	1.0		
Yes	131	2 (2)	0.1	0.02-0.9	0.048
UICC pN category					
pN0	215	8 (4)	1.0		
pN1	40	0	1.0		
pN2	7	1 (14)	3.6	0.2-81.9	0.427
pN3	2	1 (50)	15.0	1.1-205.7	0.044

**Histological grade 2**					
		Tumor recurrence		Distant organ metastasis	
		Present	HR; 95% CI *P*-value	Present	HR; 95% CI *P*-value
	420	20 (5)		17 (4)	
			**Model 1**		
Conventional tumor budding grade					
Grade 1	96	0	1.0	0	1.0

Grade 2	103	4 (4)	1.0	3 (3)	1.0
Grade 3	221	16 (7)	3.0; 0.8-10.4	14 (6)	3.2; 0.7-14.2
			0.090		0.114
Fibrotic focus					
Absent	329	6 (2)	1.0	5 (2)	1.0
Present	91	14 (15)	2.8; 1.3-6.4	12 (13)	3.7; 1.5-9.0
			0.010		0.004
Muscle invasion					
Absent	349	23 (7)	1.0	19 (5)	1.0
Present	3	2 (67)	10.4; 2.5-47.9	2 (67)	13.5; 3.0-59.7
			0.001		<0.001
Invasive tumor size (mm)					
≤20	189	2 (1)	1.0	2 (1)	1.0
>20 - ≤50	216	13 (6)	2.8; 0.6-12.6	11 (5)	1.5; 0.3-7.0
			0.182		0.591
>50	15	5 (33)	5.2; 1.7-15.6	4 (27)	4.5; 1.3-15.6
			0.003		0.017
			**Model 2**		
Proposed tumor budding grading system					
Grade I	41	0	1.0	0	1.0
Grade II	260	5 (4)	1.0	10 (4)	1.0
Grade III	51	12 (24)	5.4; 2.4-12.1	11 (22)	6.6; 2.8-16.2
			<0.001		<0.001
Muscle invasion					
Absent	349	23 (7)	1.0	19 (5)	1.0
Present	3	2 (67)	7.2; 1.5-13.7	2 (67)	8.4; 1.8-38.8
			0.007		0.006
Invasive tumor size (mm)					
≤20	189	2 (1)	1.0	2 (1)	1.0
>20 - ≤50	216	13 (6)	2.9; 0.6-13.7	11 (5)	1.7; 1.4-7.9
			0.160		0.536
>50	15	5 (33)	4.5; 1.5-13.7	4 (27)	4.0; 1.1-14.1
			0.007		0.030

Histological grade 3

Cases	No. of patients (%)							
	TR		LR		DOM		TRD	
	+	HR 95%CI P-value	+	HR 95%CI P-value	+	HR 95%CI P-value	+	HR 95%CI P-value
239	44 (18)		11 (5)		33 (14)		20 (8)	

				**Model 1**					
Conventional tumor budding grade									
Grade 1	41	3	1.0	1	1.0	2	1.0	1	1.0
		(7)		(2)		(5)		(2)	
Grade 2	51	7	1.2	1	0.9	6	1.5	4	1.2
		(13)	0.3-5.3	(2)	0.06-14.8	(12)	0.3-8.9	(8)	0.1-13.2
			0.790		0.951		0.665		0.873
Grade 3	147	34	1.7	9	2.4	25	1.6	15	2.0
		(23)	0.5-6.0	(6)	0.5-12.1	(17)	0.3-7.8	(10)	0.2-16.6
			0.431		0.273		0.570		0.540
Fibrotic focus									
Absent	134	16	1.0	3	1.0	13	1.0	6	1.0
		(12)		(2)		(10)		(5)	
Present	105	28	2.1	3	2.8	13	1.6	14	3.2
		(27)	1.1-4.0	(2)	0.7-11.3	(10)	0.6-4.0	(13)	1.1-9.0
			0.026		0.147		0.350		0.029
Adjuvant therapy									
No	10	4	1.0	1	1.0	3	1.0	1	1.0
		(40)		(10)		(30)		(10)	
Yes	229	40	0.07	10	0.1	30	0.06	19	0.2
		(18)	0.02-0.2	(4)	0.01-1.1	(13)	0.02-0.2	(8)	0.01-1.5
			<0.001		0.067		<0.001		0.111
UICC pN category									
pN0	150	17	1.0	5	1.0	12	1.0	8	1.0
		(11)		(3)		(8)		(5)	
pN1	54	16	2.6	4	2.2	12	3.0	6	0.9
		(30)	1.3-5.3	(7)	0.5-9.0	(22)	1.3-7.0	(11)	0.2-4.1
			0.009		0.272		0.011		0.977
pN2	19	6	3.7	0	2.2	6	5.1	4	3.2
		(32)	1.4-9.8		0.5-9.0	(32)	1.8-13.7	(21)	0.6-16.6
			0.007		0.272		0.002		0.165
pN3	16	5	4.1	2	5.6	3	4.3	2	1.5
		(31)	1.5-11.5	(13)	0.9-34.6	(19)	1.2-15.9	(13)	0.3-9.1
			0.008		0.065		0.031		0.664
Age (year)									
≤39	23	8	1.0	1	1.0	7	1.0	3	1.0
		(35)		(4)		(30)		(13)	
>39	216	36	0.4	10	1.1	26	0.3	17	0.6
		(17)	0.2-0.8	(5)	0.1-9.2	(12)	0.1-0.7	(8)	0.2-6.8
			0.013		0.907		0.005		0.466
Tumor-infiltrating lymphocytes (%)									

0	1	1	1.0	0	1.0	1	1.0	0	1.0
		(100)				(100)			
1-19	124	38	0.5	8	1.0	30	0.5	19	1.0
		(24)	0.04-5.2	(5)		(19)	0.03-5.9	(12)	
			0.530				0.560		
>19	76	5	0.3	3	0.9	2	0.2	1	0.1
		(7)	0.1-0.9	(4)	0.2-3.9	(3)	0.04-0.8	(1)	0.02-1.2
			0.046		0.950		0.020		0.077
Blood vessel invasion									
Absent	161	23	1.0	4	1.0	19	1.0	9	1.0
		(14)		(3)		(12)		(6)	
Present	78	21	1.5	7	4.5	14	0.9	11	1.1
		(27)	0.7-3.2	(9)	1.2-16.8	(18)	0.3-2.4	(14)	0.3-3.7
			0.321		0.025		0.812		0.884
Muscle invasion									
Absent	235	42	1.0	11	1.0	31	1.0	18	1.0
		(18)		(5)		(13)		(8)	
Present	4	2	2.9	0	NA	2	4.7	2	6.3
		(50)	0.6-15.1			(50)	1.1-21.1	(50)	1.2-32.0
			0.201				0.042		0.027
Perineural invasion									
Absent	201	32	1.0	10	1.0	22	1.0	10	1.0
		(16)		(5)		(11)		(5)	
Present	38	12	1.3	1	0.2	11	2.3	10	12.5
		(32)	0.6-2.9	(3)	0.02-1.6	(29)	0.9-5.9	(26)	2.2-15.6
			0.498		0.114		0.079		<0.001
Hormone receptor status									
Negative	115	18	1.0	5	1.0	13	1.0	10	1.0
		(16)		(4)		(11)		(9)	
Positive	124	26	0.8	6	0.8	20	0.9	10	0.3
		(21)	0.4-1.8	(5)	0.2-3.3	(16)	0.4-2.5	(8)	0.1-0.9
			0.609		0.800		0.929		0.029
				**Model 2**					
Proposed tumor budding grading system									
Grade I	33	1	1.0	0	1.0	1	1.0	1	1.0
		(3)				(3)		(3)	
Grade II	161	27	2.9	7	1.0	20	2.0	10	0.8
		(17)	0.4-22.5	(4)		(12)	0.2-17.6	(6)	0.1-7.3
			0.313				0.518		0.873
Grade III	45	16	5.5	4	2.8	12	3.2	9	3.8
		(36)	0.6-45.9	(9)	0.6-12.8	(27)	0.3-32.2	(20)	1.4-10.1

			0.120		0.192		0.313		0.008
Adjuvant therapy									
No	10	4	1.0	1	1.0	3	1.0	1	1.0
		(40)		(10)		(30)		(10)	
Yes	229	40	0.09	10	0.1	30	0.04	19	0.2
		(18)	0.03-0.3	(4)	0.01-1.2	(13)	0.001-0.2	(8)	0.01-1.7
			<0.001		0.073		<0.001		0.124
Tumor-infiltrating lymphocytes (%)									
0	1	1	1.0	0	1.0	1	1.0	0	1.0
		(100)				(100)			
1-19	124	38	0.3	8	1.0	30	0.3	19	1.0
		(24)	0.02-3.4	(5)		(19)	0.03-4.4	(12)	
			0.322				0.401		
>19	76	5	0.2	3	1.0	2	0.05	1	0.1
		(7)	0.1-0.8	(4)	0.2-4.3	(3)	0.001-0.9	(1)	0.02-1.2
			0.016		0.995		0.049		0.069
Age (year)									
≤39	23	8	1.0	1	1.0	7	1.0	3	1.0
		(35)		(4)		(30)		(13)	
>39	216	36	0.4	10	1.2	26	0.3	17	0.7
		(17)	0.2-0.9	(5)	0.1-9.6	(12)	0.1-0.8	(8)	0.2-2.9
			0.018		0.884		0.015		0.628
UICC pN category									
pN0	150	17	1.0	5	1.0	12	1.0	8	1.0
		(11)		(3)		(8)		(5)	
pN1	54	16	2.8	4	2.3	12	2.7	6	1.2
		(30)	1.4-5.8	(7)	0.5-9.9	(22)	0.5-13.8	(11)	0.2-8.0
			0.005		0.270		0.226		0.870
pN2	19	6	3.5	0	2.3	6	3.7	4	2.8
		(32)	1.3-9.0		0.5-9.9	(32)	0.6-21.4	(21)	0.3-26.7
			0.011		0.270		0.172		0.368
pN3	16	5	5.0	2	4.6	3	2.7	2	1.2
		(31)	1.8-14.0	(13)	0.7-28.9	(19)	0.3-22.8	(13)	0.1-15.7
			0.002		0.104		0.370		0.893
Blood vessel invasion									
Absent	161	23	1.0	4	1.0	19	1.0	9	1.0
		(14)		(3)		(12)		(6)	
Present	78	21	1.6	7	5.6	14	0.9	11	1.3
		(27)	0.7-3.3	(9)	1.5-20.6	(18)	0.4-2.6	(14)	0.4-4.5
			0.267		0.010		0.904		0.638
Perineural invasion									

Absent	201	32	1.0	10	1.0	22	1.0	10	1.0
		(16)		(5)		(11)		(5)	
Present	38	12	1.2	1	0.2	11	2.1	10	5.4
		(32)	0.5-2.8	(3)	0.02-1.4	(29)	0.8-5.5	(26)	2.2-13.6
			0.643		0.101		0.113		<0.001
Hormone receptor status									
Negative	115	18	1.0	5	1.0	13	1.0	10	1.0
		(16)		(4)		(11)		(9)	
Positive	124	26	0.8	6	0.8	20	0.9	10	0.4
		(21)	0.4-1.8	(5)	0.2-3.8	(16)	0.4-2.4	(8)	0.1-0.9
			0.593		0.817		0.855		0.044

HR, hazard ratio; CI, confidence interval; TR, tumor recurrence; LR, local recurrence; DOM, distant-organ metastasis; TRD, tumor-related death; +, present

Supplementary Table 5 Analyses to identify the prognostic power of the conventional tumor budding grade in cases of invasive carcinoma of no special type of the breast (Overall)

**Univariate analyses**					
	Cases	TR (%)	p-values	TRD (%)	p-values
	855	79		26	
Peripheral tumor budding (PTB)					
Grade 1	183	3 (2)		1 (0.6)	
Grade 2	208	13(6)	0.022	4 (2)	0.233
Grade 3	464	63 (14)	0.003	21 (5)	0.059
P for trend			<0.001		0.005
Intra-tumor tumor budding (ITB)					
Grade 1	225	10(4)		4 (2)	
Grade 2	218	5 (2)	0.335	1 (0.5)	0.261
Grade 3	412	64 (16)	<0.001	21 (6)	0.004
P for trend			<0.001		0.006
**Multivariate analyses**					
		HR for trend	95 % CI for trend		p for trend
**Tumor recurrence**					
PTB grade (1, 2, 3)		1.9	1.1–3.0		0.015
ITB grade (1, 2, 3)		1.7	1.1–2.7		0.011
**Tumor-related death**					
PTB grade (1, 2, 3)		2.0	0.9–5.0		0.098
ITB grade (1, 2, 3)		1.6	0.8–3.2		0.215

TR, tumor recurrence; TRD, tumor-related death; HR, hazard ratio; CI, confidence interval.
